# The structural history of Eukarya

**DOI:** 10.1126/sciadv.aeh2524

**Published:** 2026-10-28

**Authors:** Diandra Daumiller, Qiuzhen Li, Patrick Bryant

**Affiliations:** ^1^ The Department of Molecular Biosciences, The Wenner-Gren Institute. Stockholm University, Svante Arrhenius väg 20C, 114 18 Stockholm.; ^2^ Science for Life Laboratory, Solna 171 65, Sweden.

## Abstract

Comparative genomics has traditionally relied on sequence-based markers, yet the global evolutionary landscape of protein architecture remains largely unexplored. We present the Structural History of Eukarya (SHE), a structural similarity-derived phylogeny from all-vs-all structural comparisons on the proteome level of 1542 eukaryotic species, encompassing nearly 300 trillion pairwise protein alignments. This proteome-scale analysis reveals a bipartite model of eukaryotic evolution: a rigid *Strict Core* dominated by cytoskeletal architecture, supporting a more plastic *Operational Engine* centered on translational machinery. We identify lineage-specific accelerations of structural evolution in species such as birds and ants that are decoupled from proteome expansion. Finally, we demonstrate that proteome-wide structural fidelity enables the functional annotation of uncharacterized proteins and a data-driven framework for model organism selection, replacing heuristic choices with quantitative matching to human biological processes. SHE is freely accessible as an interactive portal.

## INTRODUCTION

Historically, reconstructing the Tree of Life has relied on comparing a select group of highly conserved molecular markers, such as ribosomal RNA (rRNA) (*1*). While this approach yields high-confidence phylogenetic trees for closely related organisms, its utility is constrained by the omission of vast evolutionary data contained within the broader genome. This reliance is further complicated by heterotachy, varying evolutionary rates across different genes and lineages, which introduces significant uncertainty as the divergence between organisms increases. Such limitations in sequence-based phylogeny do more than obscure ancestral relationships; they compromise the reliability of critical applications, including phylogenomic-based gene function annotations (*2*).

To overcome these hurdles, the three-dimensional (3D) structure of proteins has emerged as a more robust indicator of deep evolutionary history. Protein architecture is remarkably stable; because the biophysical requirements for folding are so stringent, structural cores evolve 3 to 10 times more slowly than their primary amino acid sequences (*3*). This stability makes structural phylogenetics a powerful tool for resolving ancient evolutionary splits that sequence data can no longer accurately track (*4*). Recent research demonstrates that structure-based models consistently outperform sequence-only methods across all levels of divergence, from distantly related proteins to those more closely aligned (*5*). The versatility of this approach has already been validated through the successful reclassification of rapidly evolving viruses (*6*) and the study of the evolution of insects (*7*).

Despite its theoretical advantages, large-scale structural phylogenetics has historically been bottlenecked by two formidable challenges: a scarcity of experimentally determined structures and the prohibitive computational cost of structural alignment (*8*, *9*). For decades, the structural universe remained largely inaccessible, as the vast majority of proteins lacked high-resolution models, and the resources required to compare those that did were immense. However, a recent technological shift has effectively dismantled these barriers. The emergence of high-accuracy AI models, most notably AlphaFold2 (*10*), has democratised structural biology by generating hundreds of millions of high-quality protein structures. This specific tool has populated the AlphaFold Protein Structure Database (AFDB) and effectively resolved the long-standing data deficit (*11*).

Complementing this influx of data, Foldseek has circumvented the computational burden of alignment by translating complex 3D geometries into a simplified “3Di” sequence alphabet (*12*). This innovation transforms resource-intensive structural comparisons into rapid, sequence-like alignments. Finally, the logistical challenge of managing these massive datasets has been addressed by Foldcomp, a high-performance compression format that allows for the efficient storage and rapid retrieval of millions of structures (*13*). Together, these advancements have transitioned structural phylogenetics from a niche speciality into a scalable, proteome-wide methodology.

Harnessing these recent breakthroughs in structure prediction and high-throughput alignment, we present an all-against-all structural analysis of complete eukaryotic proteomes. Our study establishes a robust metric of proteome similarity that accounts for both the structural conservation of individual protein folds and variations in overall proteome size. This metric allows us to construct the Structural History of Eukarya (SHE), a comprehensive and high-resolution eukaryotic similarity embedding derived from structural data. Beyond reconstructing ancestral lineages, SHE introduces a novel framework for quantifying the rate of structural proteome evolution across the eukaryotic tree. By providing the complete dataset as an interactive, open-access resource, we offer a lens through which to examine the evolutionary forces that have sculpted the diversity of eukaryotic life.

## RESULTS

### The structural similarity of eukaryotic proteomes

Protein structures are generally more conserved than sequences since maintaining the correct 3D fold is crucial for function, while different amino acid sequences can achieve the same fold through compensatory mutations (*3*, *14*). In addition, comparing organisms at the level of a single gene or protein excludes all other proteome information. To obtain a comparison between organisms on the structural proteome level, we aligned all structures from the AlphaFold database (*11*) in all complete eukaryotic proteomes from UniProt (*n* = 1542) (*15*) against each other using Foldseek (1.2 million proteome alignments, ∼300 trillion protein-protein alignments between 24.3 million unique proteins). This constitutes the largest protein structural comparison to date (to our knowledge) and required vast computational resources and novel, highly efficient pipelines that combine the FoldComp-MMseqs-Foldseek ecosystem (*12*, *13*, *16*, *17*). From the structural hits, we define the proteome similarity as

$$\text{Proteome similarity}\left(i,j\right)=\sum_{k=1}^{N}S_{k}$$ (1)

*S*_*k*_ is the highest Foldseek structural bit score (*12*) (the top hit) obtained from searching protein *k* from proteome(*i*), consisting of proteins *k*..*N*, against proteome(*j*). All proteomes (*i* = 1, 2, .., n-1, *j* = *i* + 1, .., n) are compared with each other. Each target protein in proteome(*j*) is restricted to a single optimal match to prevent redundant scoring. The result is that proteomes of different sizes are penalised, and large proteomes with high similarities are favoured, as these will accumulate significantly higher total bit score support. We define the proteome distance as the inverse of the similarity

$$\text{Proteome distance}\left(i,j\right)=\text{log}2\left(\frac{1}{\text{Proteome similarity}\left(i,j\right)}\right)$$ (2)

The logarithm with base 2 is used to balance the difference between highly similar and dissimilar proteomes, ensuring that both large and small proteome distances can be handled jointly. The resulting proteome distances were used to construct a proxy for the evolutionary history of protein structures across eukaryotic proteomes (Fig. 1, Materials and Methods). The reconstructed tree of the protein structural history of Eukarya enables studies of evolutionary rates across proteomes, proteome-wide structural comparisons and the possibility to investigate the evolution of key cellular functions in a more complete context, all of which we present below. Importantly, all alignment scores are precomputed and stored, providing a valuable resource for efficient structural comparison across proteomes without expensive re-computation of pairwise relationships at any level. One caveat is that of proteome annotation completeness, which can impact the pairwise proteome distances. We explore these limitations below and offer a web server alternative to take this into account when analysing organisms with missing annotations.

**Fig. 1.**
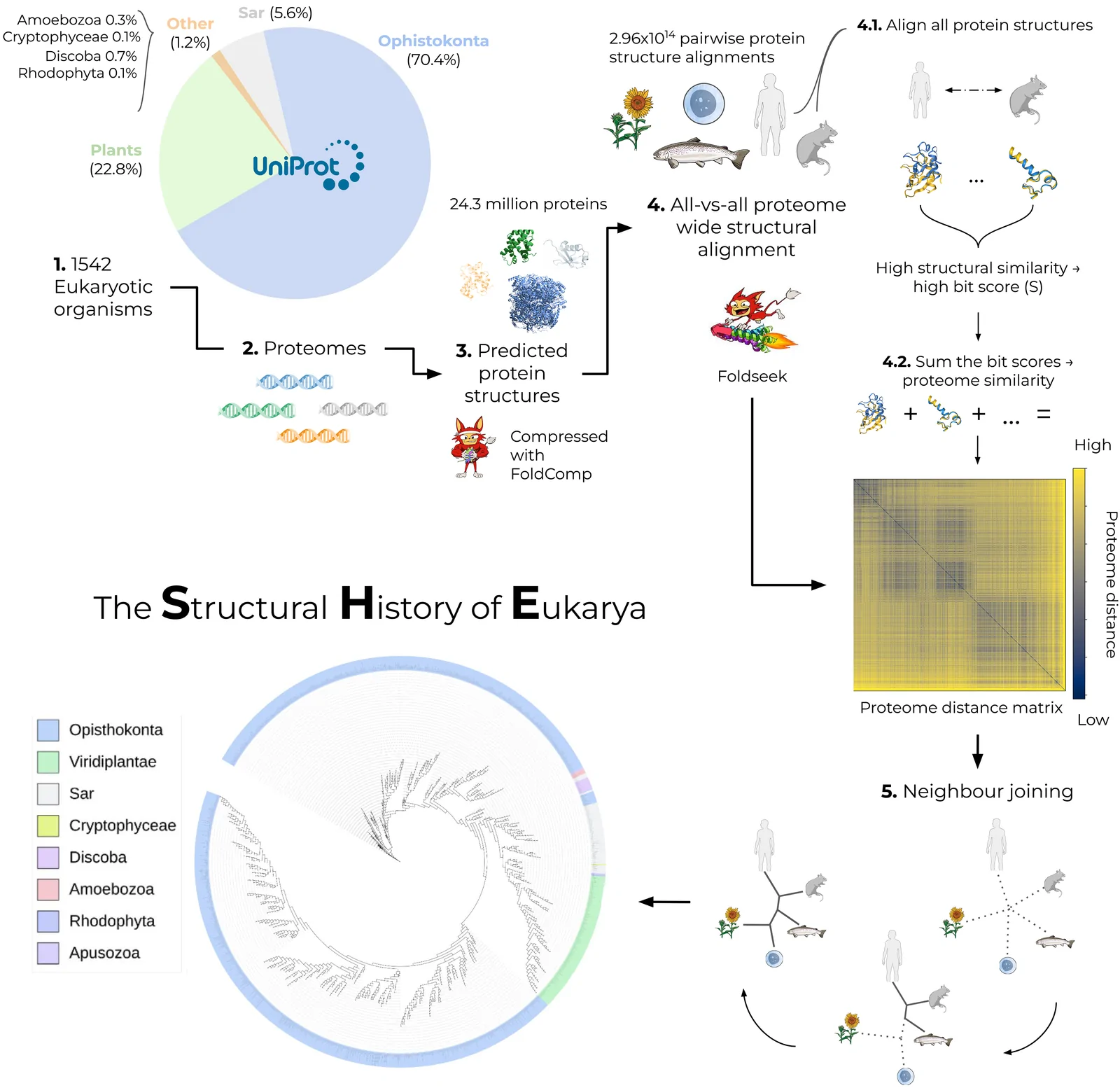
The Structural History of Eukaryotic Proteomes: a proteome-wide structural analysis workflow. To reconstruct evolutionary relationships at the structural level, we developed a high-throughput pipeline starting with (**1**) selecting Eukaryotic organisms and (**2**) extracting all eukaryotic proteomes from UniProt annotated as highly complete (*n* = 1542 organisms, median gene retention 96%, Fig. 9C). (**3**) Predicted structures for these proteomes were retrieved from the AlphaFold Database (AFDB) and compressed using FoldComp to build proteome-specific structural databases. (**4**) We then performed an all-against-all structural alignment using Foldseek, comparing every protein in each proteome against every protein in all others (**4.1,** exemplified by the human vs. gorilla comparison). This resulted in 1,188,111 proteome-level alignments comprising 2.96 × 10^14^ protein-protein alignments across 24,333,776 unique proteins. A Proteome Distance Matrix (**4.2**) was generated by aggregating the highest-scoring bit scores for each protein pair (using the inverse bit score sum, Eq. 2). (**5**) Finally, a eukaryotic tree reflecting the structural similarities between the proteomes was constructed from this matrix using neighbor-joining with bootstrapping. The circular dendrogram visualizes these relationships, with major taxonomic groups highlighted: Opisthokonta (blue), Viridiplantae (green), SAR (Stramenopiles, Alveolates, Rhizaria; pink), Discoba (rose), Amoebozoa (purple), Rhodophyta (indigo), Cryptophyceae (lime green), and Apusozoa (violet).

### The structural evolution of protein sets

#### Data-driven model organism selection

Model organisms are traditionally selected based on historical convention, experimental convenience, or comparisons of individual marker genes, approaches that may overlook broader functional conservation across biological systems. Recent advances in protein structure prediction and large-scale structural search create the opportunity to compare organisms at the level of complete protein networks, but performing such analyses remains computationally prohibitive. SHE addresses this challenge through nearly 300 trillion precomputed structural alignments spanning 22.4 million proteins across 1542 eukaryotic species. This enables rapid, proteome-wide comparisons and provides a novel quantitative framework for model organism selection. For example, identifying an optimal model for a human signalling pathway would ideally require mapping all proteins in that pathway against all proteins in every candidate species and integrating these similarities into a global measure of functional conservation, an analysis rarely feasible in practice. With SHE, this search space has already been resolved: users can select a functional protein set from any indexed organism and instantly compute a network-level structural similarity score across candidate species, transforming model selection from a heuristic process into a scalable, data-driven analysis.

##### Case study: The fanconi anaemia pathway

As an example, we provide a quantitative ranking of model organisms for the study of the Fanconi Anaemia (FA) pathway (Fig. 2). The query included key players in the pathway, FANCD2 and FANCI, which form a central hub to recruit downstream factors to the DNA interstrand crosslink (ICL) site (*18*), as well as the regulatory kinase CHK1 and the E3 ligase scaffold CUL1, which work with FBXL12 to mediate the degradation of FANCD2 during oncogene-induced replication stress (*19*). As expected, the pathway architecture is exceptionally well-conserved in *Mus musculus* (bit score = 25,999), reinforcing its status as the primary mammalian model for FA research. In contrast, pathway scores decline sharply in non-vertebrate models; *Drosophila melanogaster* and *Saccharomyces cerevisiae* yielded significantly lower scores (2790 and 341, respectively). These computational results align closely with established biological literature, as the canonical FA pathway is known to be absent or highly divergent in flies and yeast, which utilise alternative ICL repair mechanisms (*20*). Interestingly, while the vertebrate *Xenopus tropicalis* (bit score = 9073) retains a functional version of the pathway, *Caenorhabditis elegans* (bit score = 7094) exhibits a comparable score, suggesting its potential as a streamlined model for investigating specific structural components of the FA machinery. Another case study can be found in fig. S1, where we show the same possibility for human Circadian Rhythm proteins (CLOCK, PER1, BMAL1, CRY1).

**Fig. 2.**
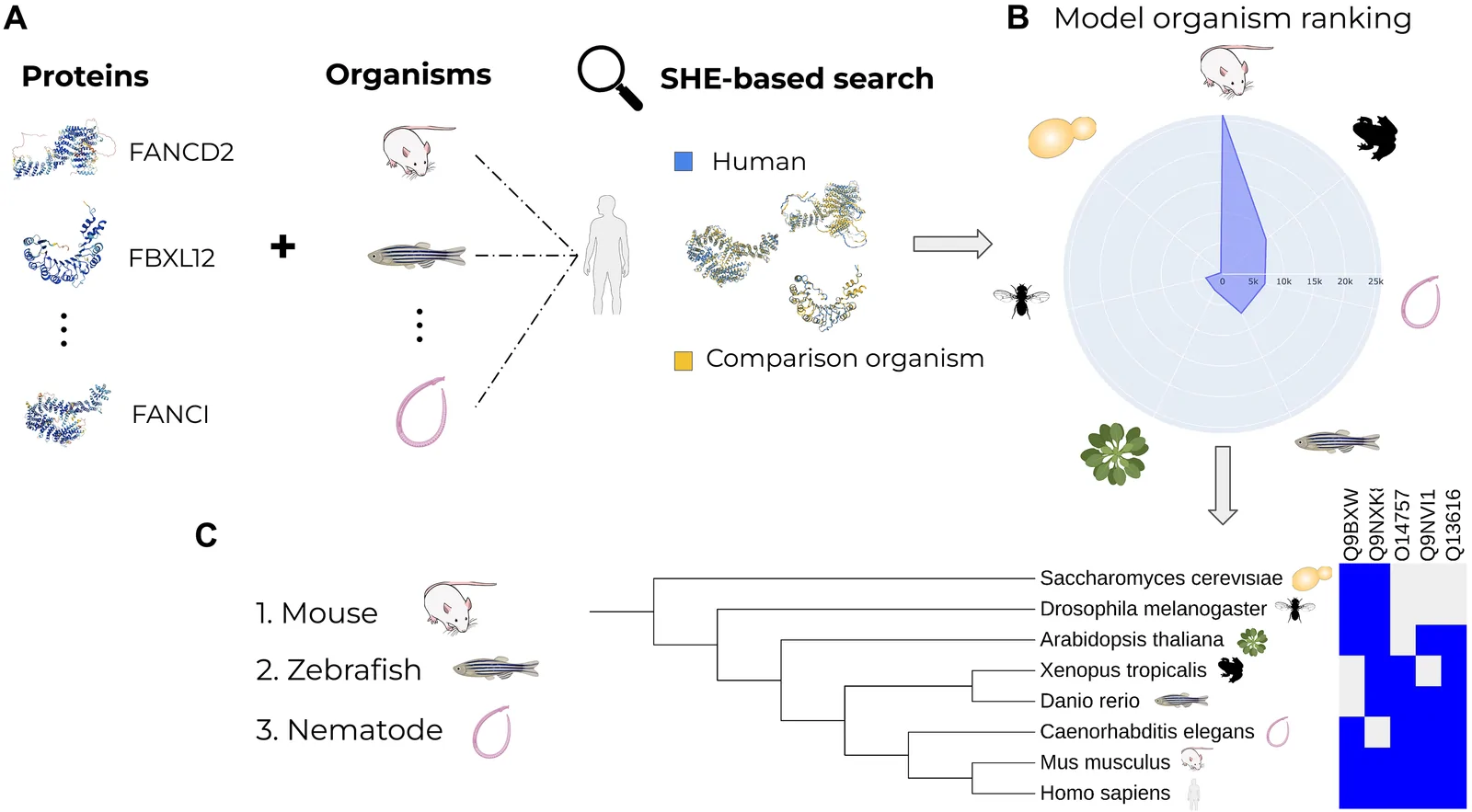
Quantitative ranking of model organisms for the Fanconi Anaemia pathway using SHE. (**A**) Schematic workflow of the SHE-based search strategy. Key human FA pathway proteins (FANCD2, FANCI, FBXL12, CHK1 and CUL1) were used as query inputs to identify structural homologs across diverse eukaryotic organisms. Pairwise structural alignments are evaluated to compare all proteins in the FA pathway across target proteomes. Rather than relying on traditional single-gene or linear sequence alignments, the SHE pipeline evaluates the cumulative pairwise structural similarity of the entire pathway to capture coordinated network conservation. (**B**) Model organism ranking based on cumulative structural similarity scores. Organisms are scored and ranked according to the sum of top-hit Foldseek bit scores across the pathway proteins. *Mus musculus* ranks highest, demonstrating the strongest preservation of overall pathway architecture, whereas non-vertebrate lineages display significantly lower cumulative scores, marking a degradation or divergence of the structural network. (**C**) Top-ranked model organisms, contextualisation, and pathway conservation profile. Left: Identification of the top three optimal model systems (*M. musculus - mouse*, *Xenopus tropicalis - Frog*, and *Caenorhabditis elegans - Nematode*). Center: Representative tree illustrating the relationship of the evaluated species. Right: Conservation heatmap indicating the presence (blue) or absence (grey) of specific human FA structural homologs across the target proteomes.

#### Structural evolution of protein families

By grouping all the analysed proteins by structure similarity (bit score > 173, e-value<10^−5^), 1,335,026 families of protein structural homologs were obtained. Although SHE is designed as a proteome-level resource, its architecture allows for the extraction of protein structure family-specific comparisons without the need for *de novo* structural alignment. By “pruning” the global SHE species tree to retain only those lineages sharing a specific structural homolog, users can visualize the evolutionary trajectory of distinct protein families (Fig. 3A). Moreover, by rebuilding the SHE tree for protein structural family sets (instead of full proteomes), the shared or divergent structural evolution of protein sets can be studied (Fig. 3B).

**Fig. 3.**
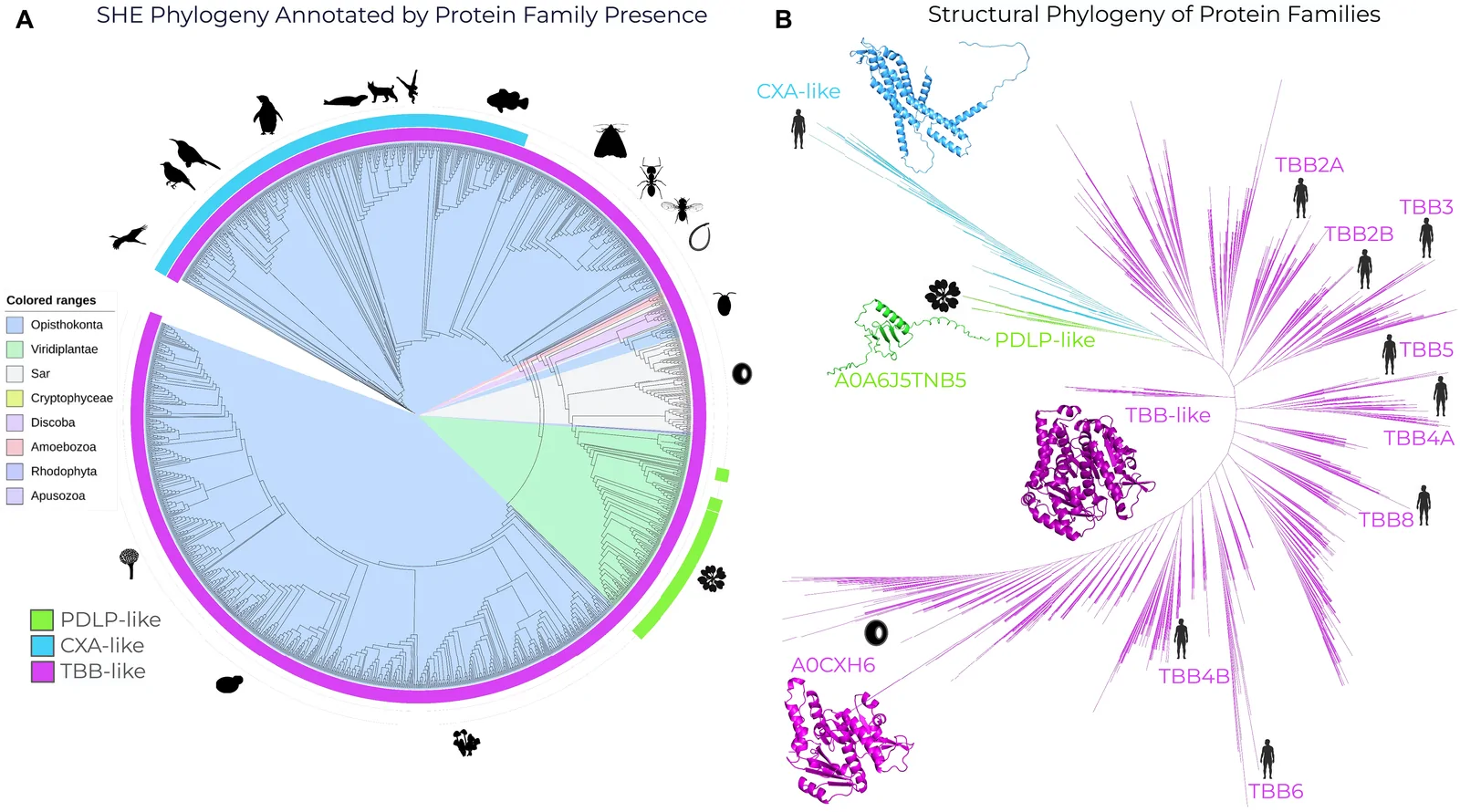
Structural similarity tree of three protein structural homolog families. (**A**) Complete SHE tree with branches colored by major clades. Outer rings denote the presence (colored) or absence (white) of three distinct protein structural homolog families across eukaryotic species: Tubulin B (TUBB; purple), Gap junction protein (CXA; blue), and Plasmodesmata-located protein (PDLP; green). (**B**) Extracted SHE structural similarity tracking the evolutionary trajectory and structural relationships among the TUBB-like, CXA-like, and PDLP-like protein sets (*n* = 100 bootstraps). Tree branches reveal differential sub-clustering based on fine-grained structural homology, with distinct human TUBB variants highlighted. The shared evolutionary root of the functional analogues CXA and PDLP is indicated, along with the bifurcation of the PDLP-like family into Plasmodesmata-located protein-like and Gnk2-homologous domain-containing proteins. Putative functional assignments based on structural clustering are highlighted for two uncharacterized proteins: A0CXH6 (*Paramecium tetraurelia*, magenta) clustering within the TUBB branch, and A0A6J5TNB5 (*Prunus armeniaca*, lime) clustering within the Gnk2-homologous domain branch.

To demonstrate the sensitivity of this capability, we analyse at once the evolution of three protein structural sets (families): Tubulin B (TUBB), Gap junction protein (CXA) and Plasmodesmata (PDLP) (Fig. 3). The TUBB family exhibits a ubiquitous distribution across Eukarya, consistent with its essential role in eukaryotic cellular physiology (*21*). Its structural similarity tree captures the full complexity of the evolution of this protein family, clustering widely conserved homologs by small differences in the chain types. The CXA and PDLP families perform similar functions of cell-cell connection and trafficking in vertebrates and plants, respectively. Thus, their shared root is not surprising. The contrast between similar gene sets that diverge structurally provides a detailed view of evolution.

This feature is useful for the identification of uncharacterized protein functions based on the structural similarity of protein *sets*, a largely unexplored capability (Fig. 3B). To show the possibility of applying this methodology to the putative functional identification of uncharacterized protein functions, the uncharacterized proteins A0CXH6 (from *Paramecium tetraurelia*) and A0A6J5TNB5 (*Prunus armeniaca*) are highlighted. Given their clustering with TUBBs and PDLPs, respectively, it is possible to infer their putative function by structural similarity.

### Evolutionary rates across proteomes

SHE enables the precise analysis of evolutionary rates of structural change across eukaryotic lineages. We find that the tempo of structural proteome evolution is highly variable across kingdoms and branching points. To quantify these dynamics, we defined the rate of proteome change as

$$R_{\text{proteome}}=\frac{\mathrm{PD}\left(i,i+1\right)}{\mathrm{PD}\left(i-1,i\right)}$$ (3)

where *PD(i, i +* 1) is the tree distance of proteomes *i* and *i* + 1 and *PD(i* − 1*, i*) is that of proteomes *i* − 1 and *i* in the order of the obtained tree directed towards the edge nodes. An R_proteome_ > 1 signifies an acceleration in structural divergence along that specific lineage, whereas an R_proteome_ < 1 indicates a deceleration or structural stabilisation relative to its ancestral baseline.

When examined at the kingdom level, the distributions of these rates show significant variation (Fig. 4, B and C). While baseline rates generally remain low across most lineages, Opisthokonta, SAR (Stramenopiles, Alveolates, Rhizaria), and Viridiplantae all exhibit extreme outliers with change rates reaching the thousands, suggesting periods of rapid evolutionary restructuring within relatively short time frames. (Note: The Apusozoa and Cryptophyceae lineages were represented by a single species, precluding distribution analysis). Such variation is a well-known phenomenon in molecular evolution, often linked to biological factors like generation time or metabolic rate (*22*), but has not previously been characterized at the global proteome level.

**Fig. 4.**
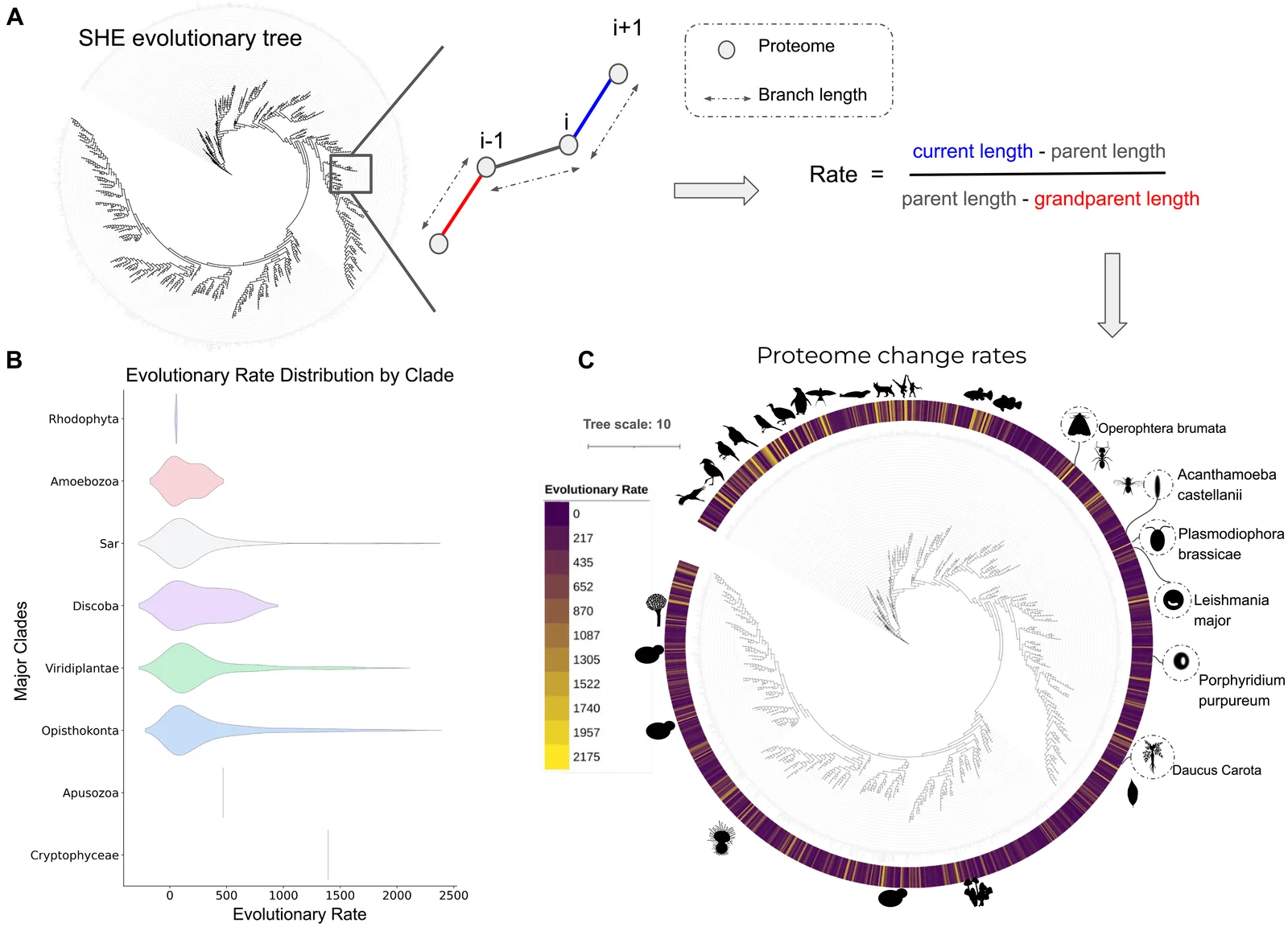
Evolutionary rate measured as the rate of proteome structural change. (**A**) Schematic of the evolutionary rate calculation. The rate of evolution for a given branch is quantified as the ratio of branch lengths between successive nodes (*i − *1*, i, i + *1). As defined in Eq. 3, this metric compares the magnitude of the most recent structural divergence (blue segment) against the preceding interval (red segment), providing a measure of evolutionary acceleration (R > 1) or deceleration (R < 1). (**B**) Distribution of evolutionary rates by clade. Violin plots visualize the density of evolutionary rates across the major eukaryotic clades: Opisthokonta (blue), Viridiplantae (green), SAR (pink), Discoba (rose), Amoebozoa(purple), Rhodophyta (indigo), Cryptophyceae (lime green), and Apusozoa (violet). Wider sections of the violin represent a higher frequency of species at that specific rate. (**C**) Global tree of evolutionary rates. The eukaryotic tree is colored according to the calculated rate of structural change, ranging from slow (dark magenta) to fast (yellow). Silhouettes highlight species with exceptionally high rates (>1500), with the top-ranking outlier labelled for each major clade (e.g., *Operophtera brumata* for Opisthokonta, *Daucus carota* for Viridiplantae). Note the distinct cluster of accelerated evolution observed within the Class Aves (birds).

The highest rates of structural evolution identified within each major clade are summarised in Table 1. Within Opisthokonta, we observed notably accelerated evolution in Aves (birds), Primates, Teleostei (bony fishes), and Formicidae (ants) (Fig. 4C). This finding, derived entirely from our structural similarity approach, corroborates the rapid evolutionary rates previously reported for groups like *Neoaves* using genomic methods [15]. Crucially, this variation in evolutionary speed appears to be intrinsic to the lineage rather than a function of data magnitude or technical artifacts. We observed no significant correlation between structural conservation and proteome size (Spearman’s ρ = 0.04) (Fig. 5A), nor did we find any meaningful relationship between our evolutionary rates and putative confounders such as proteome annotation completeness or AlphaFold structural prediction quality (pLDDT) (fig. S2). Furthermore, direct comparisons against a randomized null model confirm that the expansive rate distributions and lineage-specific bursts observed in SHE represent genuine, topology-dependent biological signals (fig. S3). Together, these results challenge simplistic models that link proteome complexity to evolutionary rate (*23*), suggesting instead that structural innovation is driven by distinct, lineage-specific evolutionary pressures.

**Table 1.** Maximum rates of structural proteome evolution across major eukaryotic clades. The table lists the species exhibiting the highest calculated rate of structural change (R_proteome_) within their respective major clades. The rate is derived from Eq. 3 and represents the ratio of structural divergence between successive nodes in the tree, where higher values indicate accelerated evolutionary shifts. Common names are provided where applicable.

Clade	Species	Common Name	R_proteome_
Opisthokonta	*Operophtera brumata*	Winter Moth	2175.3
SAR	*Plasmodiophora brassicae*	Clubroot	2105.3
Viridiplantae	*Daucus carota*	Carrot	1842.4
Discoba	Leishmania major	-	669.1
Amoebozoa	*Acanthamoeba castellanii*	-	277.0
Rhodophyta	*Porphyridium purpureum*	-	57.97

**Fig. 5.**
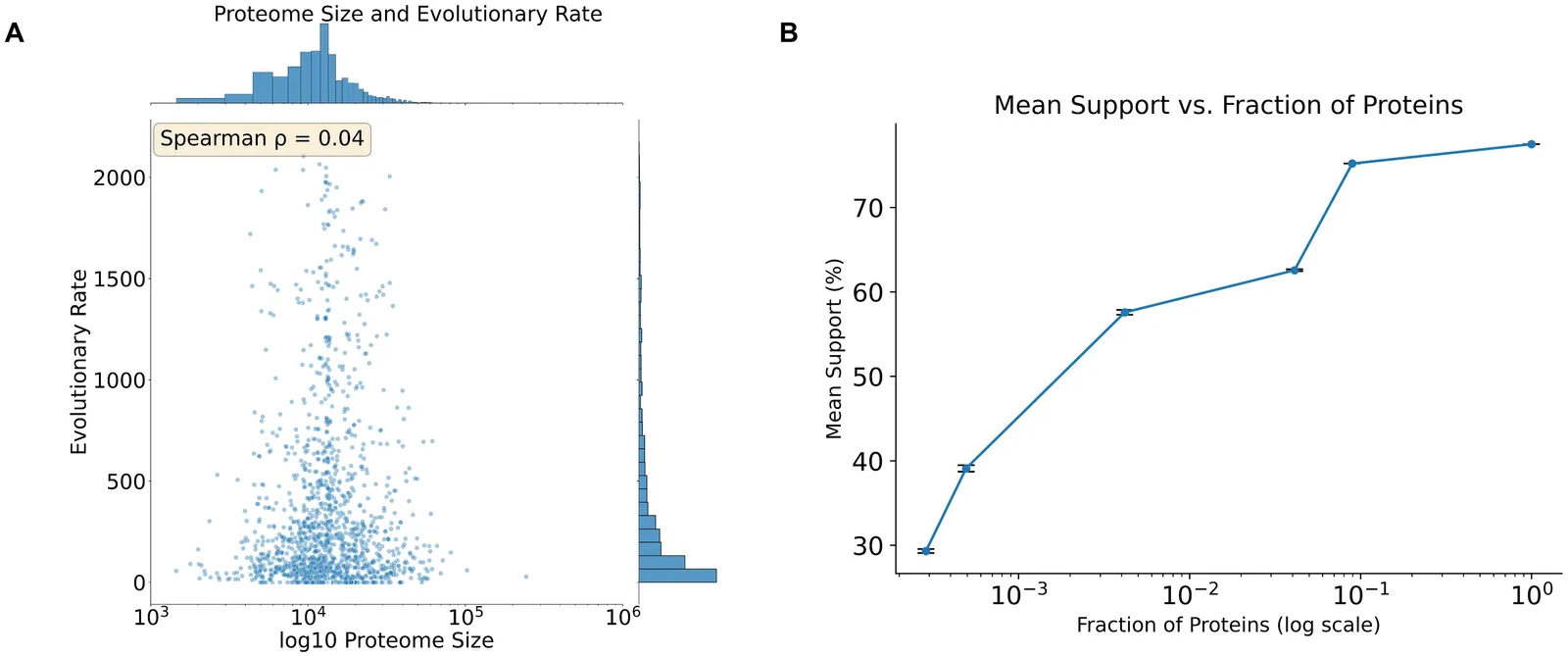
The effect of proteome size and sampling depth on evolutionary rates and structural relationships. (**A**) Correlation between proteome size and evolutionary rate. A scatter plot comparing total proteome size (log scale) against the calculated evolutionary rate (R_proteome_) for each species. Marginal histograms display the distribution of each variable. The Spearman rank correlation coefficient (ρ = 0.04) indicates no significant relationship, suggesting that structural evolutionary rates are independent of proteome size. (**B**) Impact of protein subset size on branching support. The plot illustrates the relationship between the fraction of the total proteome included in the analysis (*x*-axis, log scale) and the mean bootstrap support for the resulting structural similarity tree (*y*-axis). Data points represent the average of five independent runs with standard deviation bars. The trend confirms that increasing the volume of structural data significantly enhances the statistical robustness of the inferred tree.

### Proteome-Scale Data Enhances Resolution

A central premise of the SHE framework is that integrating structural information from complete proteomes offers superior resolution of eukaryotic relationships compared to single-gene markers. To validate this, we systematically measured how resolution scales with the volume of structural data included. We generated five distinct protein set distance matrices based on increasingly larger subsets of the most common protein families, ranging from 7000 to 2.1 million proteins. These were compared against the master structural similarity tree derived from the full dataset of 24.3 million proteins. For each subset, we constructed a neighbor-joining (NJ) tree and evaluated statistical robustness using 10,000 bootstrap replicates (Materials and Methods).

Our analysis reveals a strong positive correlation between data volume and the statistical support for the tree’s topology (Fig. 5B). A tree based on a single protein family (∼7000 proteins) yielded a mean bootstrap support of only 29.6%. In this limited tree, only 55 of 317 internal nodes (17%) achieved strong branching support (≥ 70%). As expected, increasing the structural coverage dramatically improved resolution. Mean bootstrap support rose to 39.5% with ∼12,000 proteins and reached 57.2% with a subset of 100,000. This trend continued upward, culminating in a mean bootstrap support of 77.4% for the tree constructed from the full 24.3 million protein dataset. These results demonstrate that the aggregation of proteome-wide structural signals reliably and significantly strengthens inferred evolutionary relationships.

### The core of eukaryotic proteomes

Identifying the proteins that are consistent across all eukaryotes is fundamental to reconstructing the biology of the Last Eukaryotic Common Ancestor (LECA) (*24*). Traditionally, studies aiming to define the LECA proteome have relied on sequence-based methods to identify eukaryotic homologous groups. These analyses established that the ancestor was a complex cell, possessing a core proteome of several hundred to a few thousand proteins dedicated to information processing, membrane trafficking, and metabolism (*25*).

With SHE, we can refine this view by identifying the “structural core”; a subset of proteins that maintain their 3D fold across the entire eukaryotic tree, independent of sequence divergence. We established homologous families using MCL clustering (see Materials and Methods) and filtered for those present across the vast majority of lineages. We specifically examined the ‘Strict Core’ proteome, defined as protein families conserved in ≥99% of all species. Our analysis reveals that the most evolutionarily rigid components of the eukaryotic cell are the structural and maintenance machineries (Fig. 6). The 99% Strict Core is heavily enriched for cytoskeletal elements (Structural constituent of cytoskeleton 14.4%, Fold Enrichment = 92, q < 10^−4^) and microtubule-based processes (14.7%, Fold Enrichment = 57, q < 10^−4^). This is visually exemplified by the near-perfect structural superposition of core proteins such as Actin, Tubulin, Histone H3, and HSP70 (Heat shock 70 kDa protein) between distant lineages (Fig. 6).

**Fig. 6.**
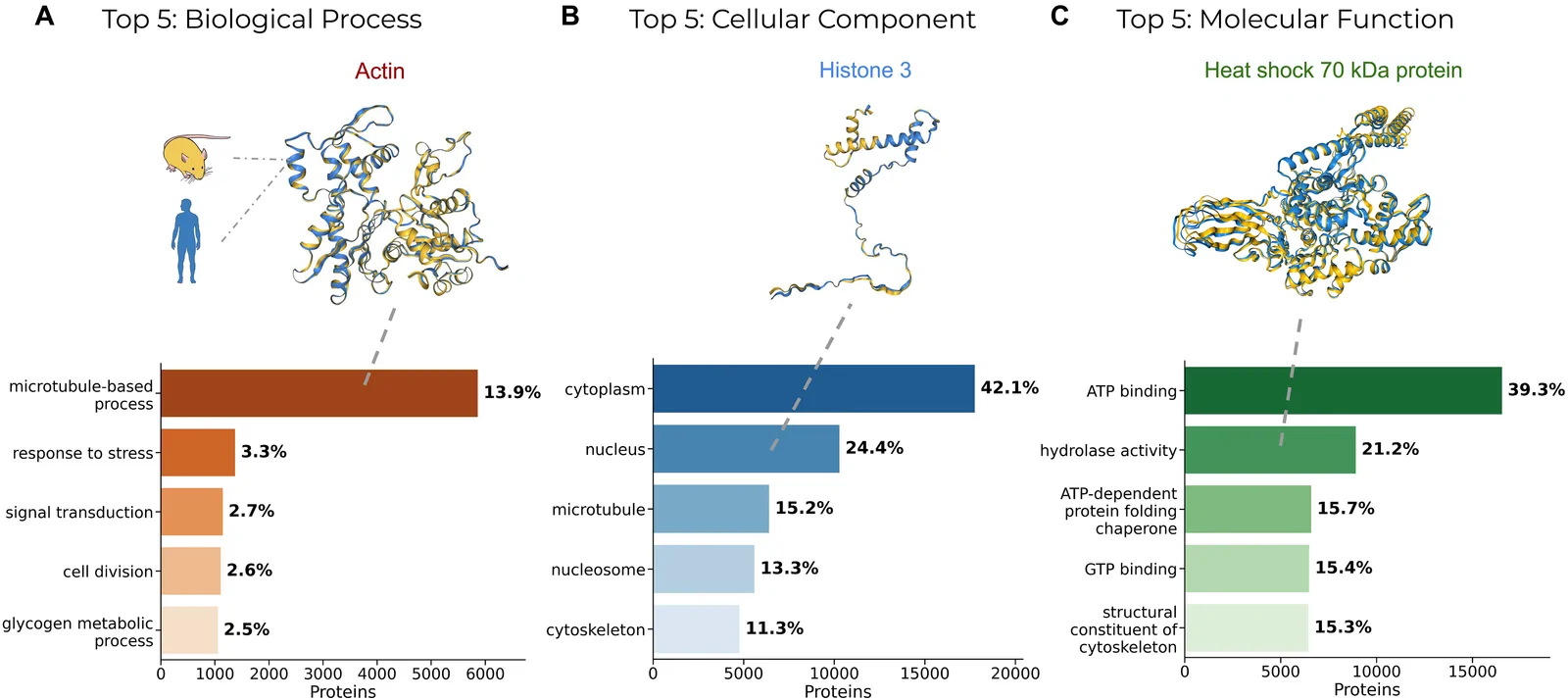
Identification and structural conservation of the core eukaryotic proteome. Gene Ontology (GO) frequency analysis was performed on the “Strict Core” protein families conserved in ≥99% of all analysed species. Bars represent the absolute number of proteins annotated with each term, with percentages indicating relative abundance within the total core dataset. Representative structural alignments between Human (blue) and Mouse (yellow) homologs are displayed above their corresponding categories to illustrate the extreme architectural preservation of the strict core. Key examples include Actin (a), Histone 3 (b), and the Heat shock 70 kDa protein (HSP70, c). (**A**) Top 5 Biological Processes: The core is dominated by structural integrity and regulation, most notably microtubule-based processes (14.7%), rather than metabolic housekeeping. (**B**) Top 5 Cellular Components: Results highlight the prominence of the nucleus (25.3%), cytoskeleton (23.8%) and cytoplasm (23.6%) as the conserved spatial framework of eukaryotic cells. (**C**) Top 5 Molecular Functions: Analysis reveals a heavy reliance on energy consumption (ATP/GTP binding) and structural quality control, such as ATP-dependent protein folding chaperones.

Interestingly, as we relaxed the conservation threshold to include families present in ≥95%, ≥90%, and ≥ 85% of species (fig. S4), the functional landscape shifted. Unlike the strict core, these broader sets are dominated by translational machinery. Already at the 95% threshold (comprising ∼900 K proteins), “translation” becomes the top biological process (8.7%, Fold Enrichment = 6.3, q < 10^−16^), and ribosome-related functions appear frequently. This suggests that while the translational apparatus is universally essential, some of its components may experience greater evolutionary flexibility or lineage-specific turnover, excluding them from the strict 99% set. In contrast, cytoskeletal and chaperone-associated proteins appear to maintain exceptionally conserved structural architectures across eukaryotes. One possible explanation is that these proteins are subject to stronger structural constraints because their functions depend on preserving precise conformational states. Alternatively, proteins involved in translation may contain domains under differing selective pressures, permitting greater structural variability while maintaining overall function. We therefore interpret this pattern as a potential large-scale organisational principle of eukaryotic proteome evolution rather than a definitive evolutionary model.

### Missing annotations limit the phylogenetic resolution in SHE

To benchmark SHE against classical markers, we compared primate phylogenies derived from 18S rRNA sequences versus our proteome-wide structural approach (Fig. 7, A and B). The SHE-derived tree demonstrates superior statistical stability, yielding consistently high bootstrap values (71–98%) compared to the variable support observed in the rRNA tree (21–100%), which often suffers from incomplete sequence sampling (e.g., missing *Mandrillus leucophaeus* data).

**Fig. 7.**
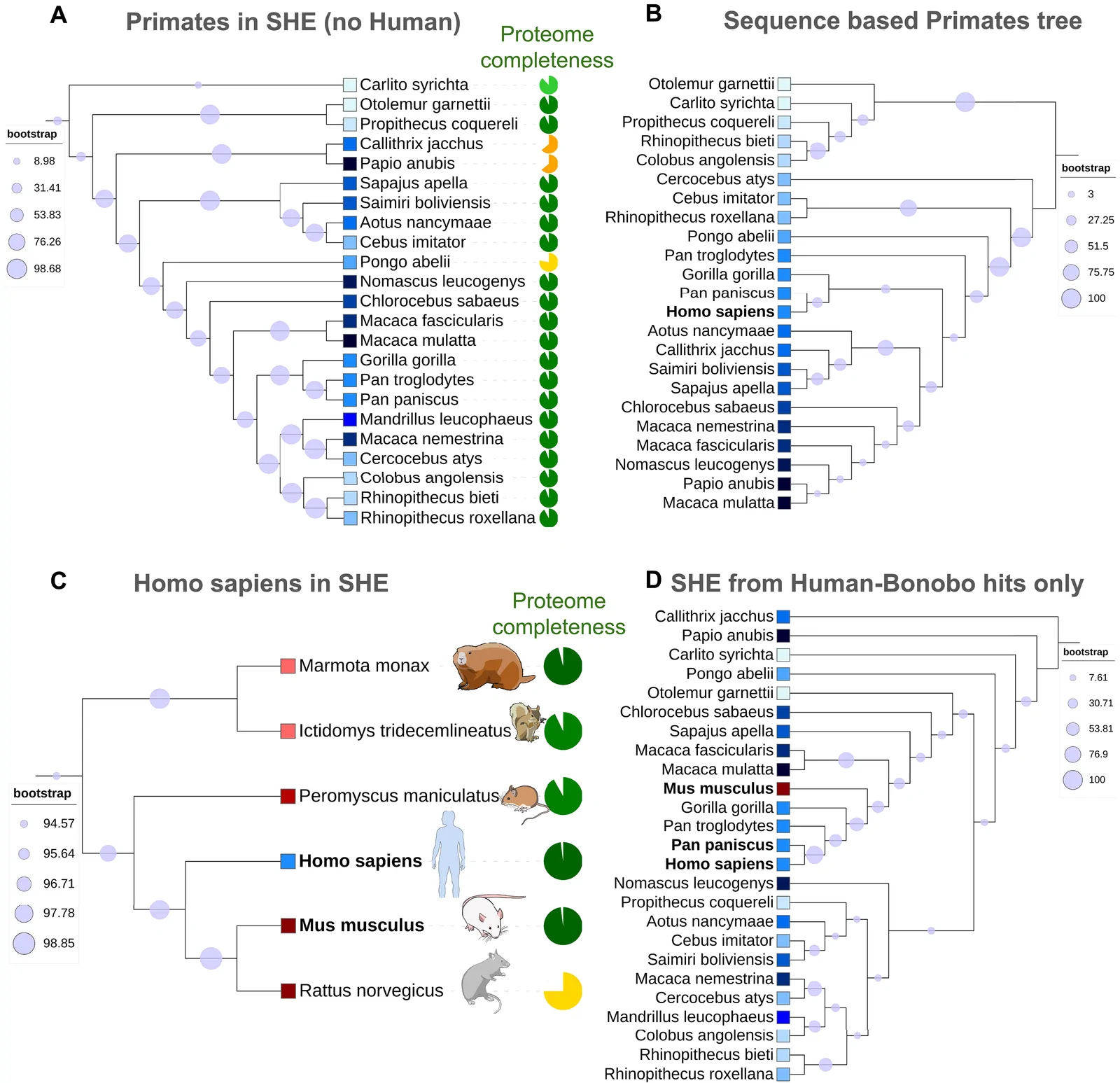
Proteome Completeness and the Structural Phylogeny of Primates. Structural similarity-derived phylogeny reveals that annotation maturity can influence local topology, serving as a diagnostic signal for database completeness. (**A**) Primate Phylogeny in SHE: A subtree extracted directly from the global SHE structural phylogeny. While bootstrap support values (violet) remain high, the topology is influenced by the proteome completeness of each species (represented by pie charts), highlighting the relationship between available structural data and branching order. (**B**) 18S rRNA Reference Tree: A benchmark phylogeny constructed from 18S rRNA sequences. Bootstrap support values (violet) show less consistency compared to the structural SHE tree. Species are color-coded by a shade of blue to facilitate a direct comparison of clustering order between sequence-based and structure-based methods. (**C**) Structural Similarity vs. Taxonomic Divergence: A detailed subtree illustrating the anomalous clustering of *Homo sapiens* (human) with rodent species. Both humans and the house mouse (*Mus musculus*) feature high proteome completeness (∼98%) and share 16,513 structural homologs. The combination of high aggregate bit-scores and near-complete structural coverage drives their proximity in the global tree. (**D**) Normalization of Annotation Bias: A SHE phylogeny pruned to a uniform set of 15,186 structural hits shared between humans and *Pan paniscus* (bonobo). By removing the impact of missing data in primate annotations, *H. sapiens* correctly clusters with its closest relatives, including *Pan troglodytes* (chimpanzee) and *Gorilla gorilla*. The inclusion of *M. musculus* (red) as an outgroup demonstrates that when annotation gaps are eliminated, structural tree distance accurately reflects evolutionary divergence.

However, while SHE provides a robust global framework, it reveals a topological discrepancy in the placement of *Homo sapiens*. Contrary to established taxonomy, which groups humans with *Pan* (bonobos and chimpanzees) and *Gorilla* (*26*), the global SHE tree clusters humans more closely with highly annotated rodents (Fig. 7C). Specifically, the prairie deer mouse (*Peromyscus maniculatus*, tree distance 0.47) and house mouse (*M. musculus*, 0.49) appear as humans’ closest relatives, while chimpanzees appear significantly more distant (9.52).

Because SHE calculates proteome distance based on cumulative alignment scores, missing proteins in less-annotated species act as “evolutionary penalties.” The *H. sapiens* and *M. musculus* proteomes are nearly complete (>98% coverage in UniProt+AFDB), whereas the *Pan troglodytes* proteome is only ∼93.9% complete, and *Papio anubis* drops to ∼64% (Fig. 7A). Consequently, humans and mice accumulate the highest density of high-scoring structural pairs, driving their artificial clustering.

To analyse this mechanism, we reconstructed the primate tree using restricted protein subsets. When the input was pruned to include *only* the 15,186 proteins shared between *H. sapiens* and *Pan paniscus*, humans correctly clustered with bonobos and chimpanzees (Fig. 7D). Similarly, utilising the 12,310 proteins shared with *P. anubis* corrected the human placement relative to baboons (fig. S5A). In these controlled analyses, *M. musculus* shifted to its correct outgroup position. This is especially evident in the tree pruned to the 2773 core proteins common to all Primates (fig. S5B). The effect of annotation completeness and strategies to adjust for this are further analysed in Materials and Methods under “Adjusting for Proteome Completeness,” concluding that not adjusting for completeness leads to a better agreement with established phylogeny.

### The SHE web server

To democratize access to the nearly 300 trillion (2.96 × 10^14^) structural alignments generated in this study, we have developed a freely accessible web platform: https://she-app.serve.scilifelab.se/. The application transforms the static data of the SHE project into a dynamic workspace for evolutionary analysis, enabling researchers to explore the structural history of eukaryotes without requiring massive local computational resources. A core feature of the server is the “Model Organism Discovery” module, which streamlines the search for optimal experimental models. By inputting any protein or pathway of interest, facilitated by guided examples, users can calculate structural conservation scores across potential model species. These rankings are derived from cumulative Foldseek bitscores and are accompanied by direct visualizations of the aligned protein structures, allowing for rapid assessment of structural fidelity.

Beyond specific pathway analysis, the platform offers a powerful environment for interactive structural similarity exploration. The global SHE tree, encompassing 1542 species, can be navigated in real-time, allowing users to zoom into major clades such as Opisthokonta, SAR, and Viridiplantae (Fig. 8). The visualization interface supports multiple layouts, including circular and linear dendrograms, and allows the tree to be dynamically colored by evolutionary rate or by the presence/absence of specific structural homologs. This functionality enables researchers to visually track gene loss events or identify evolutionary accelerators within distinct clades. Finally, to support further computational innovation, all underlying resources are made available for bulk download, including the complete matrix of Foldseek bitscores, the compressed Foldcomp databases of AlphaFold v4 predicted structures, and the Newick files for the global SHE tree and all extracted subtrees.

**Fig. 8.**
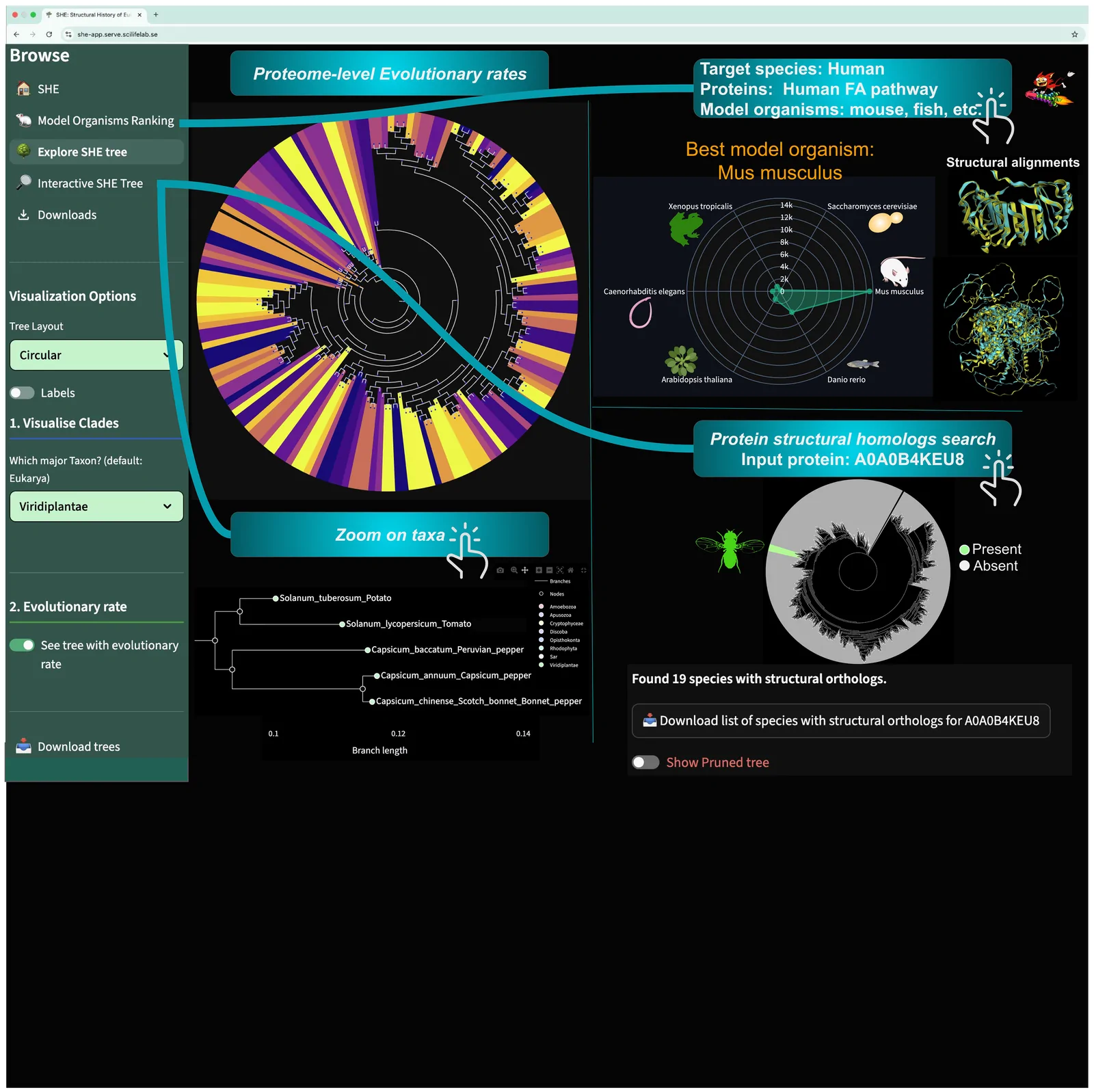
The SHE Web Server Interface. The Streamlit-based application enables real-time interaction with the structural similarity data. (**A**) Interactive Tree Exploration: The SHE app enables real-time visualization of the eukaryotic tree of life. Users can toggle visualization modes, such as coloring branches by Evolutionary Rate, and zoom into specific sub-kingdoms or species. Tooltips provide immediate access to proteome statistics, including size and calculated divergence rates. (**B**) Data-Driven Model Organism Ranking: Demonstrating the power of proteome-wide structural matching, the “Model Organism” tool accepts a set of human proteins (e.g., the FA pathway) and ranks potential model species based on structural conservation scores. This allows researchers to quantitatively validate experimental models, distinguishing between high-conservation species (e.g., *Mus musculus*) and divergent lineages. (**C**) Structural Homology and Tree Pruning: The function maps the presence/absence of structural homologs for any query protein. The interface automatically generates a “pruned” tree (right), displaying the evolutionary trajectory of specific families, such as the lineage-restricted FXNA-like protease in *Drosophila* or universally conserved “housekeeping” proteins.

## DISCUSSION

We present a high-resolution structural similarity resource derived from the all-vs-all comparison of 1542 eukaryotic proteomes. By converging rapid structural alignment (*12*) with mass-scale prediction (*10*), this work transcends the computational barriers that previously limited structural phylogenetics to small datasets. Leveraging the speed of Foldseek and the compression of Foldcomp (*13*), we integrated nearly 300 trillion structural alignments, demonstrating that proteome-scale analysis is not only feasible but essential for resolving the global evolutionary landscape beyond isolated genetic markers.

Structural similarity studies of proteomes provide a dual advantage: the stability of protein folds detects deep proteome structural evolutionary signals, while whole-proteome integration ensures robust statistical support (Fig. 7). Beyond topology, our R_proteome_ metric reveals lineage-specific accelerations, most notably within Class Aves (Fig. 4), and an analysis of potential confounders and a comparison with the rates of a null model (Fig. 5A, figs. S2 and S3) provides evidence that these rates are intrinsic biological signals rather than artifacts.

Furthermore, we resolve the eukaryotic proteome into two distinct evolutionary signatures, revealing a bipartite organisation where a rigid structural scaffold supports a plastic, yet universally essential, protein synthesis apparatus. Challenging sequence-based inferences of a metabolically complex LECA (*25*), structural analysis defines the “Strict Core” (≥99%) as primarily architectural, dominated by cytoskeletal elements and chaperones, whereas the “Operational Engine” (85–95%) is driven by translational machinery (Fig. 6). Rather than representing a definitive evolutionary progression, this bipartite configuration can also reflect differing biophysical and selective pressures: cytoskeletal proteins and chaperones may be subject to strict intrinsic constraints to preserve precise conformational states and avoid unstable evolutionary intermediates, while the translational machinery may contain highly constrained core domains alongside peripheral regions that remain permissive to structural heterogeneity and lineage-specific turnover. We therefore interpret this pattern as a large-scale organisational principle of eukaryotic proteome evolution rather than a single, definitive evolutionary model.

Selecting the right model organism is often a rate-limiting step in translational research. SHE transforms this process from a heuristic approach into a quantitative, data-driven query (Fig. 2). By pre-computing conservation scores for millions of protein pairs, our platform allows researchers to empirically rank model species based on structural fidelity. As demonstrated with the Fanconi Anaemia pathway, this capability validates established models while uncovering non-traditional candidates (e.g., *C. elegans* for specific sub-modules), thereby optimising experimental design and reducing the risk of translational failure.

A critical insight from this study is the sensitivity of proteome-wide structural similarity to data completeness. The anomalous clustering of *H. sapiens* with *M. musculus*, driven by their high annotation completeness (>98%) relative to the chimpanzee (∼94%), serves as a quantified measure of database maturity. We analyse the impact of annotation completeness (Materials and Methods, Adjusting for Proteome Completeness and Protein Abundance), showing that not adjusting for this results in a more representative tree overall (fig. S6). We also include an option on the web server to build trees with or without accounting for completeness, allowing researchers to account for this if needed.

Furthermore, while the computational scale of this initial matrix required a neighbor-Joining (NJ) approach, the future adaptation of Maximum Likelihood or Bayesian models to the structural domain will likely enhance fine-scale resolution. Nevertheless, the integrity of the global tree is evidenced by the robust recovery of kingdom-level relationships (Fig. 1). This macro-level stability confirms that a dominant proteome structural evolutionary signal persists despite localised annotation noise; had the clustering been a mere artefact of data volume, these well-established biological hierarchies would be entirely obscured. A global quality analysis confirmed that whole-proteome evolutionary relationships and tree parameters are highly robust against localised model prediction uncertainty (fig. S2).

While our exhaustive all-against-all alignment strategy establishes a comprehensive baseline, more resource-conscious hybrid approaches could be developed to lower computational overhead. For instance, future iterations might utilise cluster centroid alignments combined with exhaustive search expansions for near neighbors. However, such non-exhaustive frameworks are more complex to engineer and risk obscuring distant, low-identity homologies important for deep evolutionary resolution, and they may completely miss the lineage-specific gene duplications that SHE is designed to capture.

The future of high-resolution phylogenetics lies in the integration of structural data. As the coverage of the eukaryotic tree expands and proteome annotations improve, we anticipate that resources like SHE will redefine our understanding of deep evolutionary relationships. The framework presented here is designed for continuous growth, ready to ingest the next generation of structural predictions. By making these tools and data freely available, SHE provides a foundational platform for the community to explore the complex history of life, from the architecture of the first eukaryote to the molecular mechanisms of modern disease.

## MATERIALS AND METHODS

### Data

#### UniProt

We selected all eukaryotic reference proteomes from UniProt (*15*) on August 9 2023 (*n* = 2248). We then selected only the non-redundant ones (one proteome per organism ID) that had ≤20% missing genes according to BUSCO (The Benchmarking Universal Single-Copy Ortholog assessment tool), resulting in 1803 proteomes. All genes were downloaded in fasta format for all proteomes, and the UniProt identifiers were extracted from the fasta files. However, not all proteomes had all UniProt identifiers present in the AlphaFold DataBase (AFDB, uniprot_v4) (*11*). The median retained fraction is 95.5%, and we selected the proteomes that had at least 25% of entries in the AFDB (see Foldcomp), resulting in 1542 proteomes (86%) in the final selection.

The median proteome size is 13,898 proteins (Fig. 9A). The smallest proteome (*Ordospora colligata*, a gut parasite in shrimp) has 1810 genes, and the biggest (*Araneus ventricosus*, Orbweaver spider) has 246,021 genes. In the taxonomic prevalence (Fig. 9B), Opisthokonta is by far the most prevalent (1243 proteomes), followed by Viridiplantae (176 proteomes) and SAR (96 proteomes). The strong bias to Opisthokonta demonstrates our preference to study species related to our own and how understudied the other groupings are. This can also be seen in the proteome size distribution, where it seems like proteomes with more than 50,000 proteins are lacking.

**Fig. 9.**
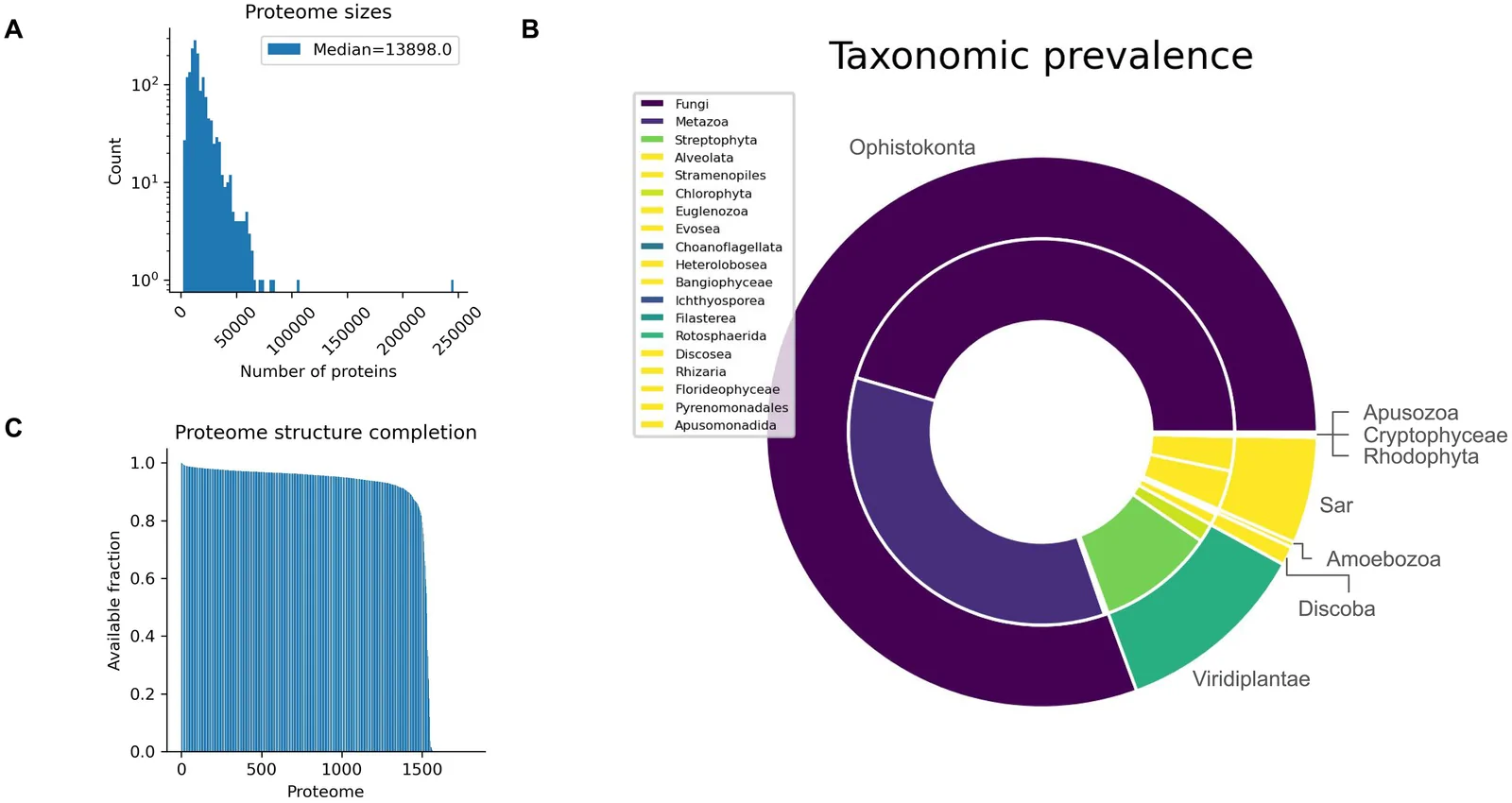
Proteome size, taxonomic prevalence and annotation completion. (**A**) Distribution of proteome sizes. (**B**) Fractions of proteomes per kingdom (text on circle) and subkingdom (list). The Ophistokonta are the most represented Eukarya, followed by plants (Viridiplantae) and SAR. Among the Ophistokonta, fungi are the most prevalent, followed by Metazoa (animals). (**C**) Available fractions of proteomes in the AFDB. Most proteomes have >90% availability.

#### Foldcomp

To save space and speed up the pairwise comparisons, we downloaded a compressed version of all predicted proteins from the AFDB uniprot_v4 (*11*) from Foldcomp (https://foldcomp.steineggerlab.workers.dev/, afdb_uniprot_v4). This database contains 214′683’829 protein structures and is 1.1 TB. We then extracted subsets of the compressed database according to the UniProt IDs from each proteome specified above. We used mmseqs2 (version ad6dfc66d7bbc4fd626fc19adf10ba587bc137c4) and the following command:

mmseqs createsubdb --subdb-mode 0 --id-mode 1 proteome_ids input_foldcomp_db output_foldcomp_db

From 1803 proteomes, 1542 could be subset with at least 25% retained proteins (median retention = 95.5%, Fig. 9C). This is because the gene identifiers and proteomes are constantly updated in UniProt, and the AlphaFold predictions are of UniProt version 2021_04. In addition, proteins with fewer than 16 or more than 2700 residues are excluded from the AlphaFold database.

#### Foldseek databases

From the proteome-specific databases created with Foldcomp and mmseqs2, we build Foldseek (version d8fab94f544fa5014274a376344c2cfd7d7530bd) databases for structural alignment using the following command:

Foldseek createdb folcomp_db Foldseek_db

### Protein structural similarity search with Foldseek

To compare all proteins between entire proteomes, we use Foldseek and the built databases of all structures in each proteome (see above) to be able to search these efficiently. Foldseek computes a structural bit score that combines the 3Di alignment and structural scores: “*We rank hits by a ‘structural bit’ score - that is*, *the product of the bit score produced by the Smith-Waterman algorithm and the geometric mean of average alignment LDDT and the alignment TM-score.”* (*12*)

For each proteome i = 1, .., n-1:

1) Search proteome j = i + 1..n with Foldseek, where n denotes the total number of proteomes. The following command was used for the search:

Foldseek search Foldseek_db(i) Foldseek_db(i+1) aln(i,i+1) <tmpDir> -e 0.01

2) Thereafter, the results (with evalue < 0.01) are formatted into a tsv:

Foldseek convertalis Foldseek_db(i) Foldseek_db(i+1) aln(i,i+1) result(i,i+1)--format-output "query,target,alnlen,evalue,bits"

3) The highest bit score for each protein in proteome i is selected as the top hit in proteome j = i + 1. This ensures the proteins’ structures are similar. Each protein in proteome j = i + 1 is used only once. This means that if two proteins have identical top hits, one will get a score of 0. This ensures differences between duplicated genomes, which are ignored by conventional phylogenetic methods.

Searching 1542 proteomes against each other (excluding self-interactions) results in [1542 × (1542–1)]/2 = 1,188,111 comparisons. Applying the same calculation to the number of proteins in each proteome results in 2.96 × 10^14^ protein alignments between the 24,333,776 unique proteins. For the initial global database sweep, an inclusive Foldseek alignment significance threshold of E-value <0.01 was chosen. Rather than acting as a highly conservative final cutoff, this threshold functions as a baseline filter to capture both tight structural matches and highly distant or novel structural homologs across deeply separated lineages, which are then further compared using more stringent criteria. 22,399,012 unique proteins successfully established at least one high-confidence, unique top structural hit across the 1,188,111 proteome-pair comparisons.

### Proteome-wide structural distance calculations

To calculate the distance between two proteomes (i.e., the proteome distance), we use all obtained top hit bit scores for all comparisons in the protein similarity calculations (*n* = 2.96 × 10^14^). Figure 10A shows the distribution of all bit scores, and Eqs. 1 and 2 in the main text specify the proteome similarity and distance, respectively. We sort the proteomes by the number of proteins and search the biggest against the smallest to capture size differences (bit score = 0 for missing matches). A distance matrix is produced by calculating all pairwise distances between all proteomes (Fig. 10B).

**Fig. 10.**
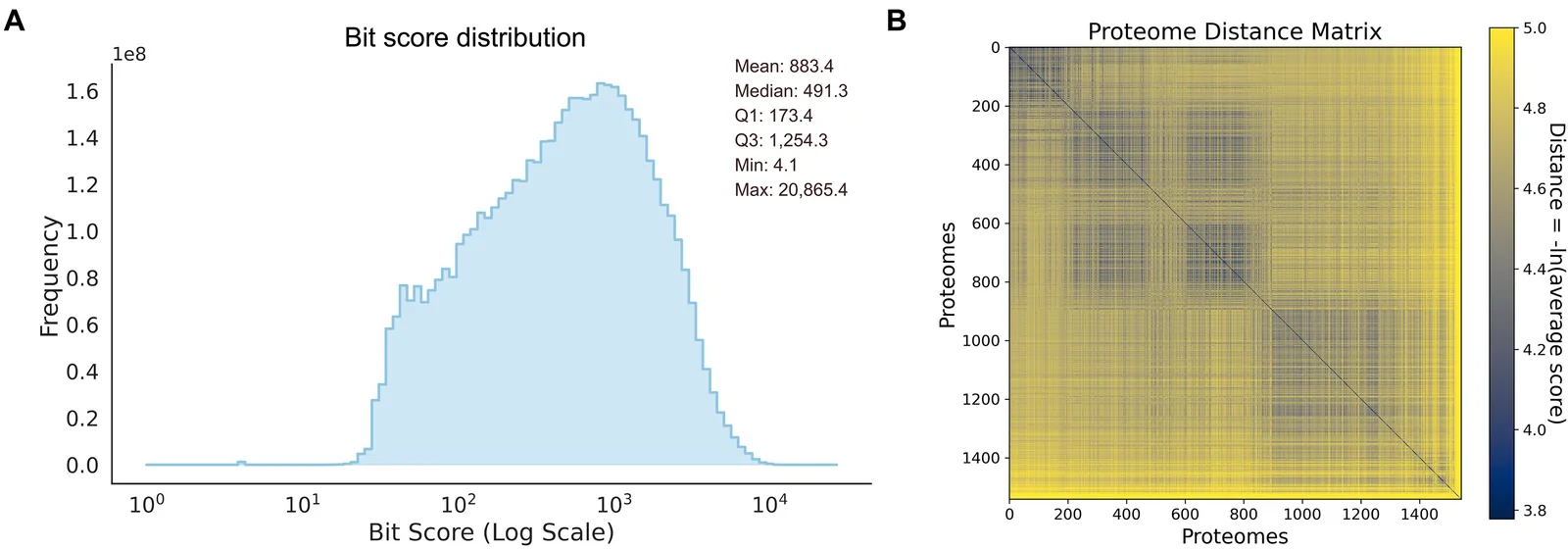
Global analysis of proteome similarity and distance. **a)** Histogram and corresponding Kernel Density Estimate (KDE) showing the frequency distribution of summed bit scores from all pairwise proteome comparisons. The x-axis shows the base-10 logarithm of the bit score sum. This is a measure of overall sequence similarity between two proteomes. The y-axis indicates the count of proteome pairs for each bit score sum. **b)** Heatmap of the pairwise evolutionary distances between proteomes. The distance, indicated by the color scale, is calculated as the negative natural logarithm (−ln) of the mean similarity score. Darker colors represent smaller distances and higher similarity, while lighter colors indicate larger distances and lower similarity. The diagonal results from the self-comparison of each proteome, having a distance of zero.

### Neighbor joining and alternative tree-building approaches

Unlike sequence-based phylogenetics, which models discrete substitution processes, our approach operates on continuous structural similarity aggregated across complete proteomes and therefore does not assume an explicit evolutionary substitution model. We use neighbor-joining [NJ (*27*)] following FoldTree (*5*) to construct the evolutionary relationship between all proteomes. This method produces accurate relationships given that the distances between the proteomes are accurate. More advanced methods, such as maximum likelihood or parsimony, exist but are not possible to apply on this scale, and it is not known what model of evolution is the most accurate. Parsimony methods assume that the smallest, simplest trees are the best, but it is possible that evolution does not work in this way at all. Instead, we choose to rely on the overall structural similarity between two proteomes and NJ (*28*).

To rigorously validate the robustness of the NJ algorithm for embedding this proteome distance space, we performed alternative tree-building benchmarks across a randomized representative subset of 100 eukaryotic species (fig. S7). Because our structural pipeline natively generates a proteome-wide distance matrix rather than a multiple sequence alignment, running traditional sequence-based Maximum Likelihood (ML) models is technically impossible. Instead, we reconstructed alternative topologies for this 100-species subset using the Fitch-Margoliash (FM) (*29*) and Minimum Evolution (ME) (*30*) criteria, two statistically grounded distance optimisation methods. All trees were built using 10000 bootstraps. Quantitative topological comparisons, calculated as the percentage of tree agreement based on Generalized Robinson-Foulds (*31*) (see below) topological agreement, revealed high congruence ranging between 90% and 94% across all three distance-based optimisation frameworks. This robust topological agreement demonstrates that the primary NJ heuristic is highly sufficient and computationally stable for large-scale structural embedding.

### Bootstrapping

A phylogenetic tree is usually bootstrapped to build a consensus tree and to obtain estimates of the confidence in the branching (*32*). As it is too computationally demanding to recompute alignments and no evidence-based model for structural substitutions in evolution exists (to our knowledge), we apply this methodology to the proteome distance matrix directly. By randomly removing pairwise relationships, we build a tree using 10,000 replicates with the Phylo package from BioPython (version 1.81) (*33*). We employed 10,000 replicates to ensure topological stability, as lower counts produced unresolved polytomies. While the count of strongly supported nodes (≥70%) converged early, the total resolution continued to improve with replicate count (fig. S8A, table S1). Specifically, 10,000 replicates resolved 1539 internal nodes, which is close to the theoretical maximum of 1541. In contrast, 1000 replicates yielded only 1525 nodes. Median bootstrap support remained consistent (∼89%) across replicates, while minimum support values converged near zero, indicating the stable recovery of distinct low-support nodes (fig. S8B, table S2).

### Normalization of the tree

To improve the visual clarity of the structural similarity tree, all branch lengths were subjected to a two-step mathematical transformation. First, the lengths were scaled to a range between 0 and 1 using min-max normalization

$$\text{Lnorm}=\frac{L-\text{Lmin}}{\text{Lmax}-\text{Lmin}}$$ (4)

Here, *L*_*min*_ and *L*_*max*_ are the minimum and maximum raw branch lengths in the tree, respectively. Second, to invert the scale and expand the differences between the shortest branches, a negative logarithmic transformation was applied to the normalized lengths to compute the final branch length, *L*_*new*_

Lnew=−0.1×In(Lnorm+0.01) (5)

This procedure was designed to enhance the visual spread between clades, with the constant 0.01 serving to avoid an undefined result for zero-length branches. The resulting tree retains its original topology, but features rescaled branch lengths optimised for clearer interpretation.

### Analyzing the similarity with previous phylogenies

Tree topological similarity was quantified using the Generalized Robinson-Foulds (RF) distance based on Mutual Clustering Information (MCI), which measures the amount of phylogenetic information shared between the split partitions of two trees, expressed in bits (*31*). To control for differences in tree resolution and size, the absolute clustering information distance was normalized against the total entropy of the splits (R packages through python subprocess: ape v5.8.1 (*34*), TreeDist v2.14.1 (*35*), TreeTools v2.4.0 (*36*)). This yielded a normalized MCI distance tightly bounded between 0 (indicating perfect topological identity) and 1 (indicating complete information divergence). The final percentage of structural topological agreement between tree pairs was computed as the complement of this distance metric (1- normalized MCI distance).

We used the Python ETE3 toolkit (*33*) to prune the consensus TimeTree of Life database (https://timetree.org/) (*37*) down to 1086 species shared (the missing taxa were either not found or TimeTree had insufficient data to place them) with SHE as a reference (root at *Acanthamoeba castellanii*). Figure S6 shows the MCI agreement of SHE with TimeTree being 75.5% compared to a randomized null model (SHE Shuffled), displaying an agreement of only 7.1%.

To assess topological agreement at each branch level, we also evaluated every internal split in the pruned SHE tree against all nodes in TimeTree, based on their species composition. For each SHE branch, we computed the MCI score against its top matching split in TimeTree. After normalization to each branch entropy, the MCI scores revealed that structural differences between SHE and TimeTree are mostly confined to terminal branches, especially in Eumetazoa, while the deep tree backbone remains mostly conserved (fig. S9A). As expected, this concordance pattern is completely lost in the randomized control comparing SHE shuffled to TimeTree (fig. S9B).

### Calculating the structural evolution of protein sets

#### Model organism selection

To identify the most suitable model organisms for the study of a specific pathway, we developed a scoring pipeline. A list of target human UniProt identifiers (for example, Q9BXW9, Q9NXK8, O14757, Q9NVI1, Q13616 for the FANCD2-dependent DNA repair, Fig. 2, or CLOCK, PER1, BMAL1, CRY1 for Circadian Rhythm, fig. S1) can be queried against pairwise proteome alignment datasets comparing *H. sapiens* (or any other target species within SHE) to a panel of candidate model organisms. For each candidate species, we calculated a cumulative bit score by summing the maximum bit scores of the identified homologs (Eq. 1). To account for bidirectional database orientation, the pipeline automatically detected and processed both forward (Target-vs-Organism) and reverse (Organism-vs-Target) alignment files.

#### Structural evolution of protein families

To extract the family-specific structural relationships, we set a strict bit score cutoff of ≥173, which aligns with the first quartile (Q1) of the bit score distribution (Fig. 10A) and effectively retains the top 75% of the most significant structural alignments. We then created a similarity graph. In this graph, each protein is a node, and an edge indicates a statistically significant structural alignment. We divided the graph into distinct protein families using a connected components algorithm from the Python library NetworkX (*38*). Each resulting component represents a group of structurally related homologs and paralogs.

We then utilised the ETE3 toolkit (*39*) to generate the tree for each isolated family (Fig. 3). We mapped the species IDs of the proteins within each family to their corresponding organism names in the global tree. To build a structural evolution tree from protein family sets, the protein family distance matrices were generated by processing homologous pair data in chunks. For every identified pair within a specific protein family, the bit score was converted to a distance metric (Eq. 2), and served as input for constructing structural evolution trees for each protein family using neighbor-joining with bootstrapping (*n* = 100 replicates).

### Evolutionary rate calculations

To quantify the tempo of structural divergence across the eukaryotic tree, we calculated a proteome-wide evolutionary rate (R_proteome_). This metric represents the ratio of structural change in a given lineage relative to its immediate ancestor, as defined in Eq. 3 (see Results, Fig. 4).

The rates were calculated by traversing the neighbor-joining tree toward the terminal nodes. For each node, the “current” branch length was compared to the “parental” branch length. To ensure the robustness of the reported accelerations and decelerations, we implemented a numerical stability filter: if the ancestral branch length (the denominator) was less than 10^−3^ structural distance units, the rate was excluded from the analysis (assigned as 0 and not used for inferring evolutionary rates, effectively NA).

This thresholding was necessary because branch lengths nearing zero indicate that the lineages are too structurally similar to calculate a meaningful ratio, and excluding them correctly identifies them as areas of the tree where the structural signal is below the resolution limit for rate comparison. All subsequent distributions and outlier analyses (e.g., the lineage-specific accelerations in *Class Aves*) were performed using this filtered dataset to ensure that high R_proteome_ values reflect genuine biological shifts rather than computational artifacts.

### Proteome-scale data and phylogenetic resolution calculations

Traditional phylogenomics relies on substitution models [e.g., Jukes-Cantor (*40*)] that assume discrete character states in DNA or amino acid sequences. However, these models do not apply to structural proteomics, where evolution is measured through continuous geometric similarity and fold conservation rather than point mutations. Because structural folds are more conserved than sequences, they can persist over vast evolutionary timescales, but they also risk signal “saturation” or noise when analysing entire proteomes.

We hypothesised that the aggregate structural signal from thousands of protein families would provide a more stable and higher-resolution evolutionary map than isolated markers. To test this and determine the minimum data threshold required for a robust structural phylogeny, we conducted an experiment utilising five nested subsets of widely distributed homologous families.

These subsets were designed with increasing scales of structural data: Level 1 (6989 proteins, 1 family), Level 2 (12,006 proteins, 2 families), Level 3 (101,674 proteins, 39 families), Level 4 (1000,112 proteins, 2,362 families), and Level 5 (2,176,597 proteins, 178,220 families) (Fig. 5B). For each subset, we generated a pairwise inter-proteome distance matrix derived from the aggregate Foldseek bit scores of the included proteins. The distance between any two proteomes was calculated using Eq. 2 with the addition of a constant factor of 2 to the denominator to ensure mathematical stability at the lower end of the bit-score spectrum. Using these matrices, we constructed phylogenetic trees via the neighbor-joining (NJ) algorithm and evaluated nodal confidence through 10,000 bootstrap replicates. This iterative scaling allowed us to quantify the gain in statistical support and taxonomic resolution provided by transitioning from single-family markers to proteome-wide structural datasets.

### The core of Eukarya and GO enrichment

To find a structural core shared by all Eukarya, we used a filtering strategy focused on structural similarities and taxonomic distribution. We compared all the proteomes in a complete structural analysis. First, we created a list of homologous protein families from hits with a Foldseek bit score over 173 and an E-value of 0.0005. Next, we grouped protein families generated via a graph clustering method, the Markov Cluster Algorithm (MCl) (*41*). We applied MCL to the weighted network of homologous protein pairs using an inflation parameter (I) of 2.0 to balance cluster granularity. To functionally characterize these families, we extracted all constituent protein UniProtKB IDs and mapped their Gene Ontology (GO) (*42*) terms (within the three categories Biological Process, Molecular Function, and Cellular Component) m using the UniProt database Gene Association File (GAF; release of June 17, 2026) (*15*, *43*). To generate a global functional profile, we performed a frequency analysis of GO terms across the entire dataset for each threshold (99%, 95%, 90%, and 85%) (fig. S4).

#### Hypergeometric test with FDR correction for determining the significance of GO enrichment

To evaluate the statistical overrepresentation of selected Gene Ontology (GO) terms across the four eukaryotic core thresholds (99%, 95%, 90%, and 85%), a one-tailed hypergeometric test was performed (fig. S4). For each top GO term, the total background population (*N*) was defined as the total number of annotated proteins within the 22.4 *M* proteins in the top hits comparisons, while the background successes (*M*) represented the global count of proteins assigned to that specific GO term. The total number of proteins (*n*) and of proteins associated with each GO term (*k*) were computed for each threshold.

The probability of observing k or more proteins associated with a specific GO term by chance was calculated using the hypergeometric survival function

$$P\left(X\geq k\right)=1-\sum_{i=0}^{k-1}\frac{\left(\begin{array}{c} M\\ i \end{array}\right)\left(\begin{array}{c} N-M\\ n-i \end{array}\right)}{\left(\begin{array}{c} N\\ n \end{array}\right)}$$ (6)

To control the inflation of Type I errors resulting from simultaneous testing across the top three terms in the three GO categories (Biological Processes, Cellular Components, Molecular Functions), raw *P* values were adjusted independently within each core threshold. False Discovery Rate (FDR) correction was performed using the Benjamini-Hochberg procedure, and the respective q-values were obtained (*44*). To quantify the magnitude of overrepresentation, fold enrichment (FE) was computed for each term within each threshold as the ratio of the observed proportion to the expected background proportion

$$\mathrm{FE}=\frac{k/n}{M/N}$$ (7)

### Global quality analysis of the SHE tree topology

To evaluate whether localised structural prediction uncertainty introduces systematic artifacts or structural topology bias at a macroevolutionary scale, we performed a global data quality control analysis across all 1542 eukaryotic proteomes. For each proteome, per-residue model confidence scores (pLDDT) were extracted from the structural repository by parsing the Cα atom records. Four global confidence parameters were computed per proteome: mean pLDDT, median pLDDT, and the stoichiometric fractions of total residues exceeding confidence thresholds of >70 (confident) and > 90 (highly confident).

To test for confounding data-driven artifacts, these four structural quality vectors were systematically regressed against primary phylogenetic metrics, including terminal branch lengths, lineage-specific structural evolutionary rates, and node bootstrap support values (fig. S2). Correlation profiles across all combinations exhibited weak trends. Specifically, structural tree branch lengths exhibit minor trends when plotted against mean and median proteome pLDDT (Spearman rank correlation coefficients ρ = 0.25 and 0.30, respectively). Although statistically significant, we find that model quality accounts for less than 9% of total branch length rank variance (median pLDDT ρ^2^ = 0.087). Therefore, while longer branches show increased point spread with occasionally lower pLDDT values, this does not impact calculated tree distances across >91% of cases. Lineage-specific structural evolutionary rates do not display a systematic correlation (ρ < 0.1) with structural disorder or lower-confidence regions, confirming that biological acceleration signals are driven by true structural divergence rather than algorithmic uncertainty. Finally, high bootstrap support remains consistently distributed regardless of the average underlying pLDDT of the proteome (ρ = 0.006 to 0.02), proving that lower-confidence predictions do not introduce topological instability into the global tree architecture. Although structure prediction uncertainty can influence local alignments, global proteome-level relationships remained robust after filtering for high-confidence models, suggesting that SHE primarily captures stable structural signal rather than prediction noise.

### Adjusting for proteome completeness and protein abundance

#### Proteome completeness

To evaluate the topological impact of variable annotation completeness across the 1542 eukaryotic proteomes (Fig. 9C), we conducted a global sensitivity analysis. First, we regressed proteome completeness against computed lineage-specific evolutionary rates and node bootstrap support values. No significant global correlations were observed (r = −0.001 and 0.077, respectively, fig. S2), indicating that varying database completeness does not systematically damp or artificially inflate calculated evolutionary parameters across the global dataset.

However, because localised missing annotations can artificially decrease cumulative structural similarity scores and misplace individual taxa (such as the artifactual clustering of *H. sapiens* with *M. musculus* due to shared high completeness relative to under-annotated sister lineages, Fig. 7 and fig. S5), we developed a joint-completeness proteome distance normalization framework. We computed a weighted proteome distance matrix where the raw distance (*D*) between any two proteomes (Eq. 2) is scaled by the product of their respective completeness fractions (*c1* and *c2*), defined as

$$D_{\text{weighted}}=\frac{D}{c_{1}\cdot c_{2}}$$ (8)

This transformation mathematically penalizes and dilutes proteome distances driven by low joint completeness.

Reconstructing the global phylogeny using this weighted distance matrix can resolve local, completeness-driven clustering artifacts. However, applying this scaling uniformly across deep macroevolutionary scales reduced the overall agreement with established phylogeny (75.5% vs 71.9% agreement according to generalised RF, fig. S6). Consequently, the unweighted structural similarity matrix is retained (and recommended) for the primary structural similarity analysis, while the completeness-weighted proteome distance matrix is deployed as a localised diagnostic option on the SHE web server to assist end-users in evaluating potential annotation-driven topological shifts.

#### Protein abundance

In addition to joint-completeness normalization, we evaluated a structural frequency-normalized proteome distance space to explicitly control for protein family expansions and redundant domain architectures across Eukarya. In this framework, the raw Foldseek bit score (*S*_*k*_ in Eq. 1) for each top-hit protein pair is normalized by dividing it by the natural logarithm of the product of their global database frequencies

$$S_{k,\text{normalized}}=\frac{S_{k}}{\text{ln}\left(f_{1}\cdot f_{2}\right)}$$ (9)

where *f*_*1*_ and *f*_*2*_ represent the total number of occurrences of the respective query and target proteins across the entire SHE database. This transformation downweights highly ubiquitous structural folds while amplifying rare, lineally informative structural matches.

To benchmark the topological robustness of our framework across these metric definitions, we computed a global tree using this frequency-normalized matrix and evaluated it against both our original embedding and the reference TimeTree of Life (fig. S6). The frequency-normalized tree shares a high 94.2% topological agreement with our original unweighted SHE tree and achieves a 75.7% agreement with the TimeTree reference framework. This robust congruence demonstrates that the macroevolutionary backbone of the SHE structural similarity space is highly stable and resistant to systemic biases introduced by localised domain duplications or family expansions.

### Primate tree construction

We compared the trees of primates between the structure-based approach (created as above) and a sequence-based approach. We applied the ETE3 toolkit (*39*) to extract the similarity relationships of primates from SHE (Fig. 7A). To construct the sequence-based phylogenetic tree of primates (Fig. 7B), we retrieved 24 primate 18S rRNA sequences from the NCBI nucleotide database (*15*, *43*). Sequences were aligned using MUSCLE (*45*). Pairwise sequence distances were then calculated using the Kimura 2-parameter (K2P) model (*46*) to generate a distance matrix suitable for 18S rRNA sequences, as it accounts for the higher frequency of transitions relative to transversions in ribosomal RNA. Phylogenetic analysis was performed using the NJ method with 10,000 bootstrap replicates to construct the tree and assess branch support. All alignments, distance calculations, and tree construction were conducted in MEGA 12 software (*47*). To reconstruct structural similarity relationships from a protein subset (i.e., Fig. 7C and fig. S5), a similar approach to the Model Organism selection was applied. We use a dataset of proteins as a reference query set (e.g. the hits between *H. sapiens* and *Pan Paniscus*). For each comparison species (i.e. all Primates), we generated a structural similarity profile by extracting the maximum bit score for each input protein against all the comparison proteomes. The resulting score vectors for all species were aggregated into a distance matrix, which was further used to construct a phylogenetic tree using neighbor joining with 10,000 bootstraps.
