# Supplementary material for: The structural history of Eukarya
Source: Sci Adv. 2026 Oct 28;12(44):eaeh2524. doi: 10.1126/sciadv.aeh2524 (PMC13715714; doi:10.1126/sciadv.aeh2524)
Supplement: Supplementary file 1 [file sciadv.aeh2524_sm.pdf]

Science Advances
AAAS

Supplementary Materials for

The structural history of Eukarya

Diandra Daumiller *et al*.

Corresponding author: Patrick Bryant, patrick.bryant@scilifelab.se

Sci. Adv. **12**, eaeh2524 (2026)
DOI: 10.1126/sciadv.aeh2524

This PDF file includes:

Figs. S1 to S9
Tables S1 and S2

Supplementary Information

Supplementary Figures

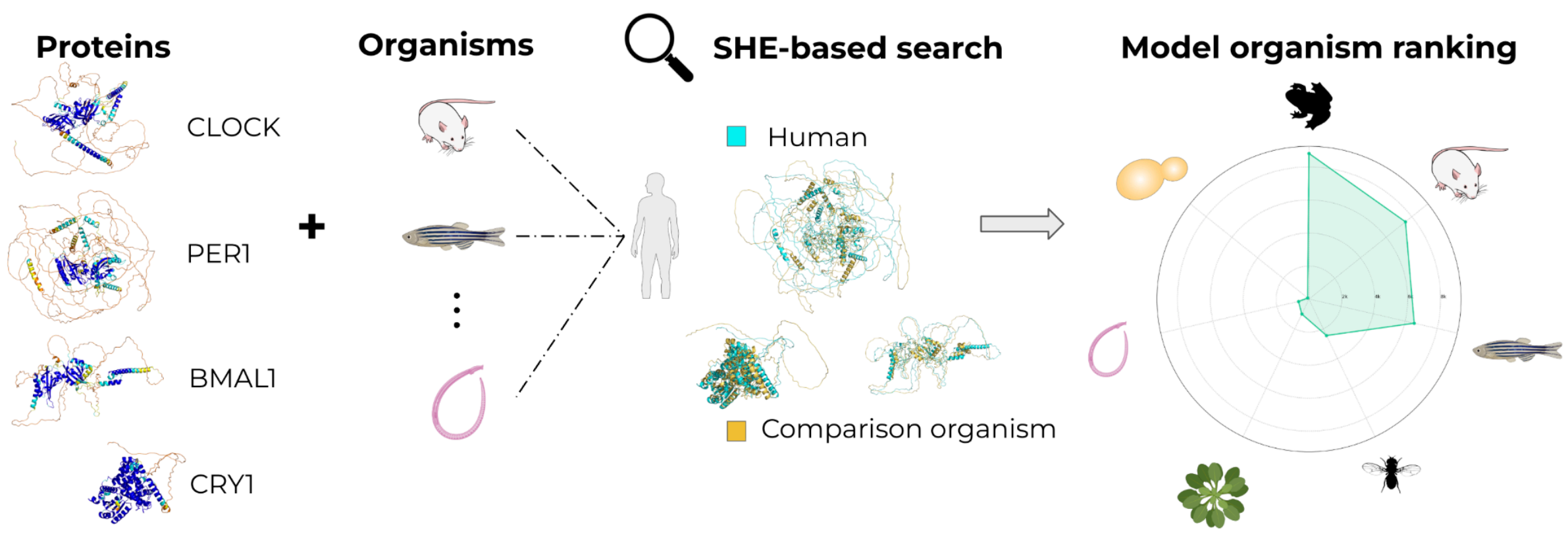

Supplementary Figure 1: Quantitative ranking of model organisms
Schematic workflow of the SHE-based search strategy. Key human Circadian Rhythm proteins (CLOCK, PER1, BMAL1, CRY1) were used as query inputs to identify structural homologs across common eukaryotic model organisms. Pairwise structural alignments were evaluated and ranked to compare all proteins in the pathway across target proteomes. Even though classically studied in *Drosophila melanogaster, Xenopus tropicalis* is the best model organism within the query organisms, followed by *Mus musculus*, highlighting the core structural and functional differences in the Circadian Rhythms between human and *Drosophila melanogaster.*

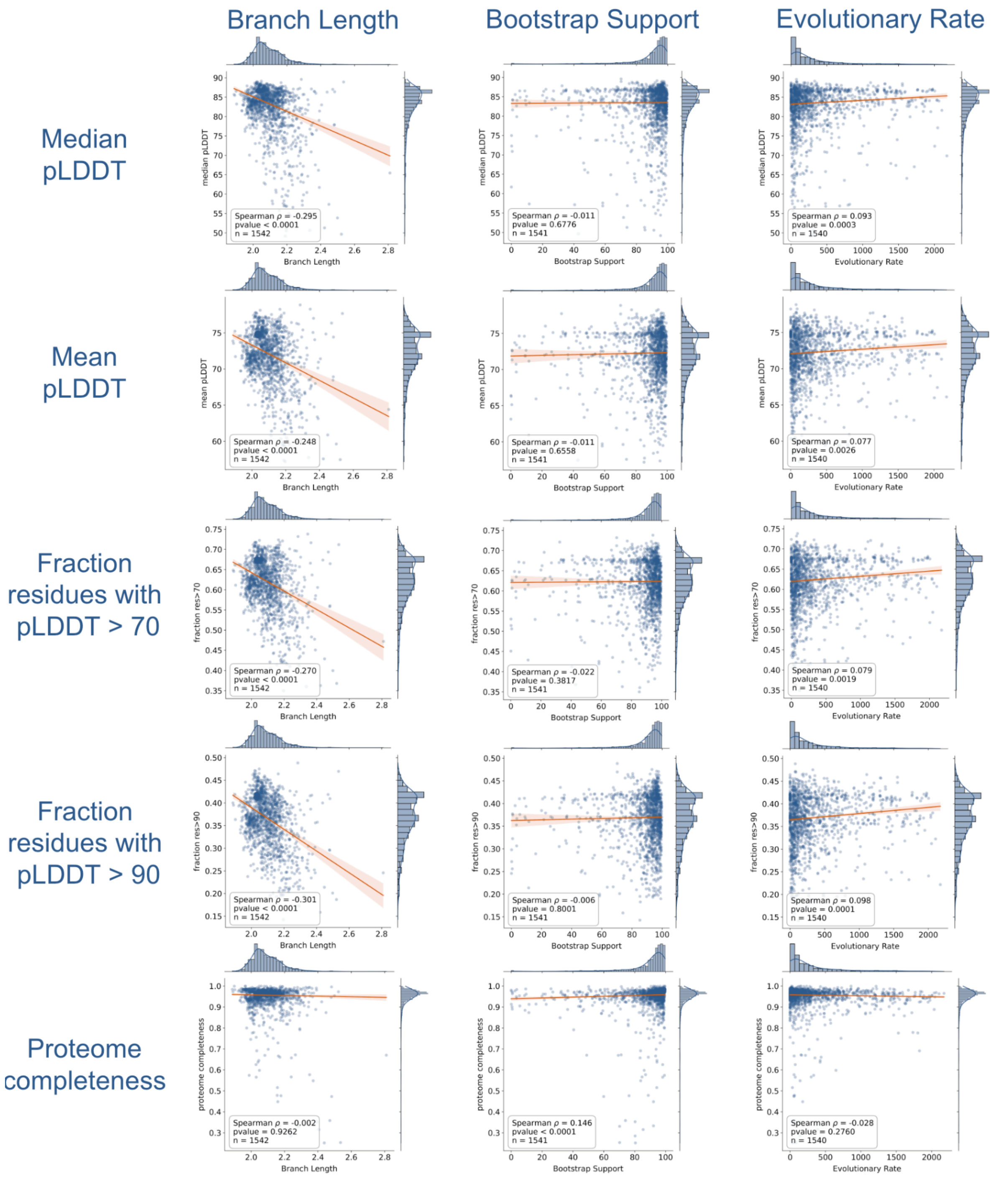

Supplementary Figure 2. Proteome completeness and pLDDT do not affect SHE tree topology. SHE tree topology (branch length, evolutionary rate and bootstrap support) was correlated to proteome completeness (retained ratio), mean proteome pLDDT, median proteome pLDDT, and fraction of residues (fraction res) with pLDDT above 90 or 70 at the proteome level. The points represent each proteome and the distributions are outlined on each axis. The straight lines represent the best least squares fit with shaded 95% confidence intervals. No strong correlation can be observed for any comparison. For each correlation, the Spearman ρ, the p-value and the number of species (n) are reported. Note that the evolutionary rate cannot be calculated for the first two species at the root (hence n=1540),

and the bootstrap support cannot be calculated for the first species in the tree (hence n=1541).

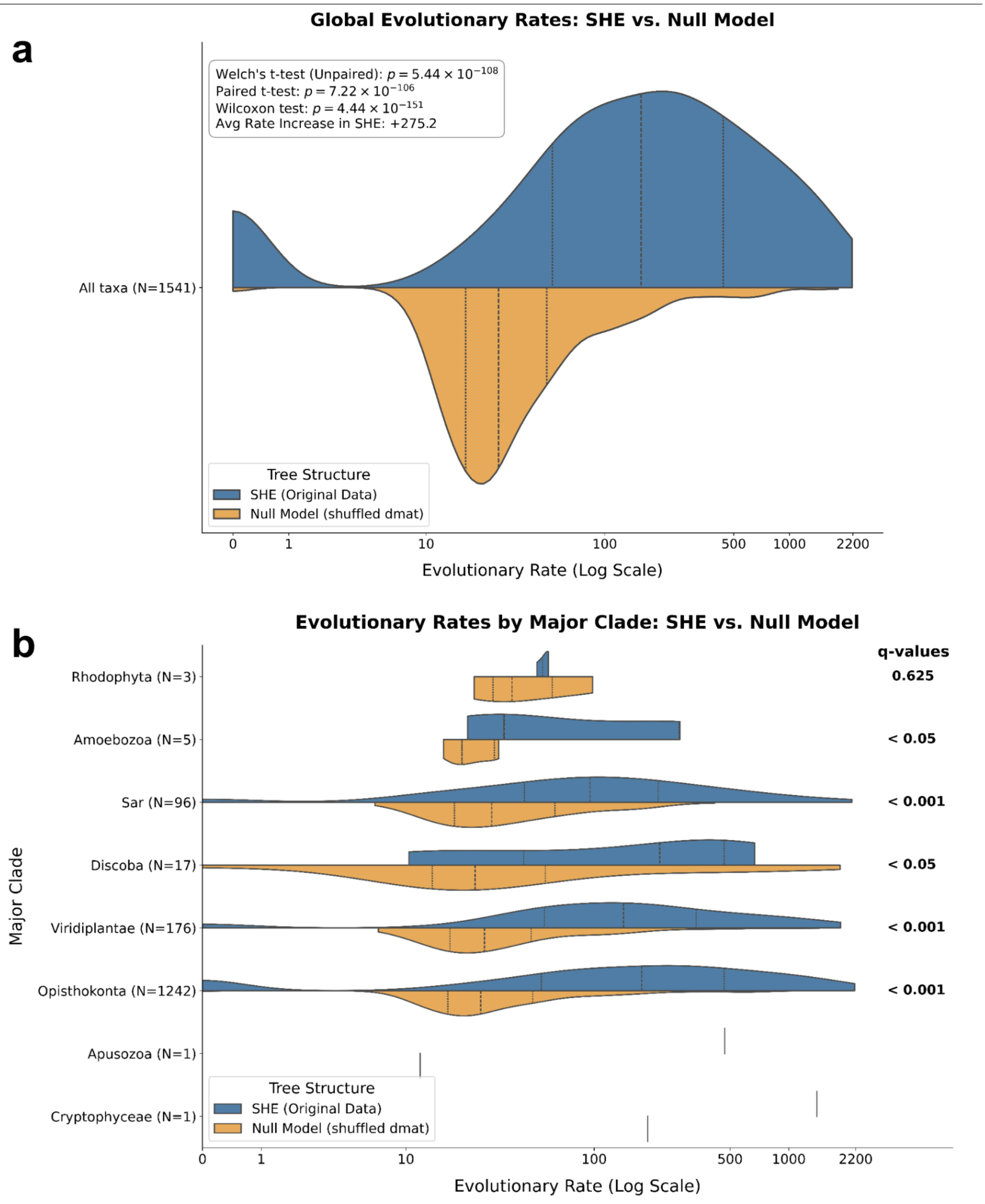

Supplementary Figure 3. Comparison of SHE-derived evolutionary rates against a randomised null model. a) Split-violin plot contrasting the empirical distribution of evolutionary rates across SHE (blue) versus a randomised null model generated by shuffling the distance matrix (orange). The distributions obtained from SHE are consistently shifted towards higher evolutionary rates (+275.2 on average: mean of the paired evolutionary rate

per species), independently of the type of statistical test performed: Welch's t-test (unpaired, unequal variance), paired t-test (global means comparison), and Wilcoxon test (paired, medians comparison). **b)** Split-violin plot contrasting the empirical distribution of evolutionary rates across taxonomic kingdoms under the true phylogenetic architecture (SHE, blue) versus the randomised null model generated by shuffling the distance matrix (orange). The distributions obtained from SHE are consistently shifted towards higher evolutionary rates. FDR-adjusted p-values (q-values) are displayed per major clade. The statistical significance of the Not all clades display statistically significant differences compared to the null model. The clade to which the fastest evolving organisms (ants and birds) belong to (Ophistokonta) is significantly different. This structural divergence suggests that the observed rate spikes are genuine biological signals dependent on authentic phylogenetic topology rather than numerical instability.

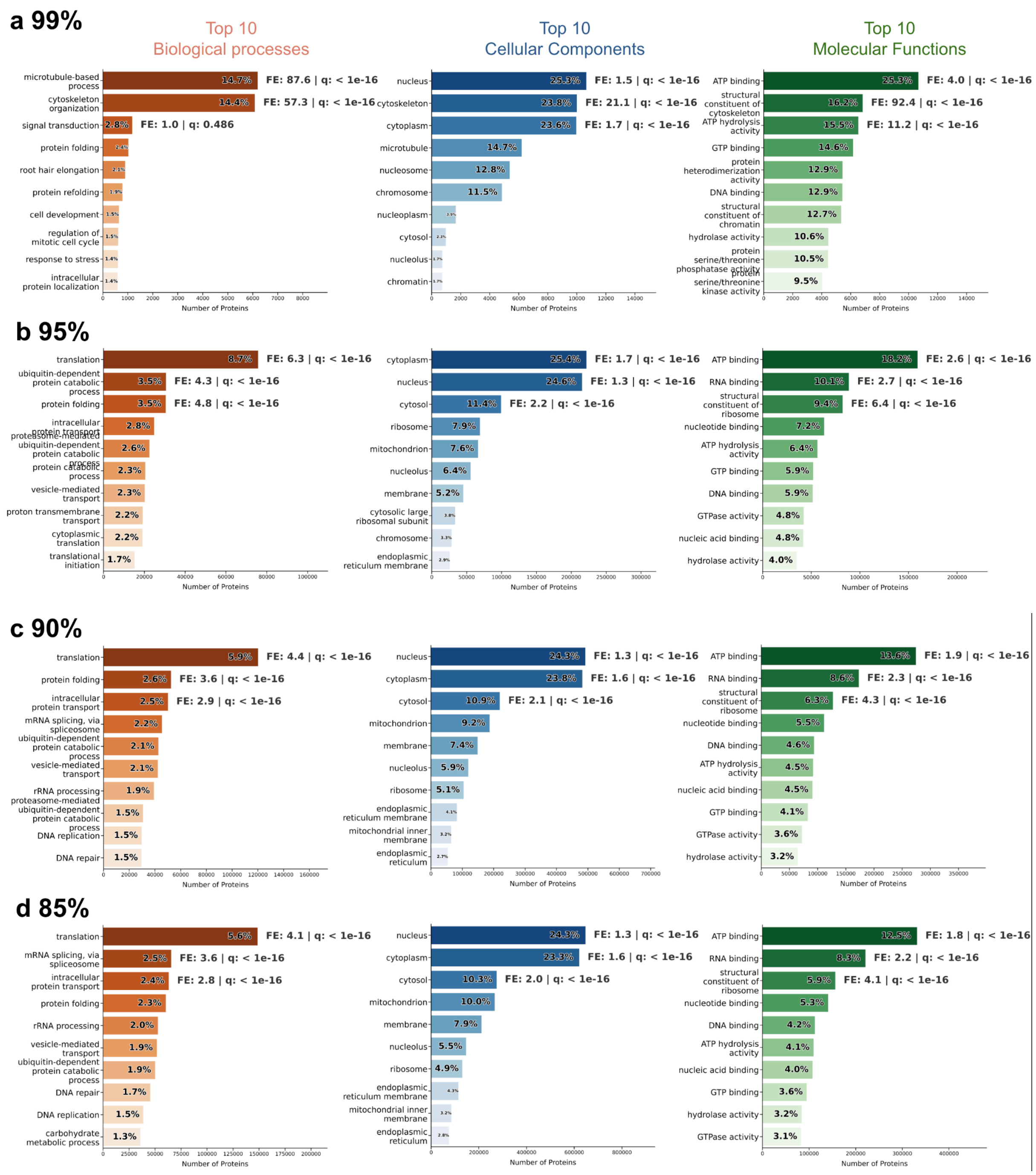

Supplementary Figure 4. Functional Stratification and Scaling of the Eukaryotic Structural Core. Gene Ontology (GO) enrichment analysis (from GAF) of human non-intraspecies paralogous proteins across varying structural conservation thresholds. Bar lengths represent the absolute number of proteins annotated with each term, while the inner labels indicate the percentage relative abundance of that term within the total core dataset for each specific threshold. For the top 3 GO terms in each plot, the fold enrichment (FE) and q-values (Benjamini-Hochberg FDR correction) are reported on the right side of the bar. Overall, each top GO term is significant and enriched, except for signal transduction. **a)** The Strict Core (≥99% conservation; n = 42,128): the top 10 GO terms capture essential

regulatory and architectural functions. Biological processes are dominated by cytoskeleton organisation, while ATP binding represents the primary molecular function. **b)** The Intermediate Core (≥95% conservation; n = 874,787): at this relaxed threshold, the functional landscape shifts toward the emergence of translational machinery. Consequently, the ribosome becomes an important Cellular Component (7.9%), together with the Molecular Function structural constituent of the ribosome (9.4%). **c)** The Operational Core (≥90% conservation; n = 2,022,223): this tier is dominated by universal housekeeping machinery. Translation (5.9%) and protein folding (2.6%) remain the top Biological Processes, as well as RNA binding functions (8.6%). **d)** The Broad Core (≥85% conservation; n = 2,659,960): At the most relaxed threshold, the functional profile stabilises with increased volume. Translation (5.6%) and protein transport (2.4%) continue to define biological processes. Ligand binding is the dominant molecular function, reflecting the broad enzymatic and cofactor requirements of the core proteome.

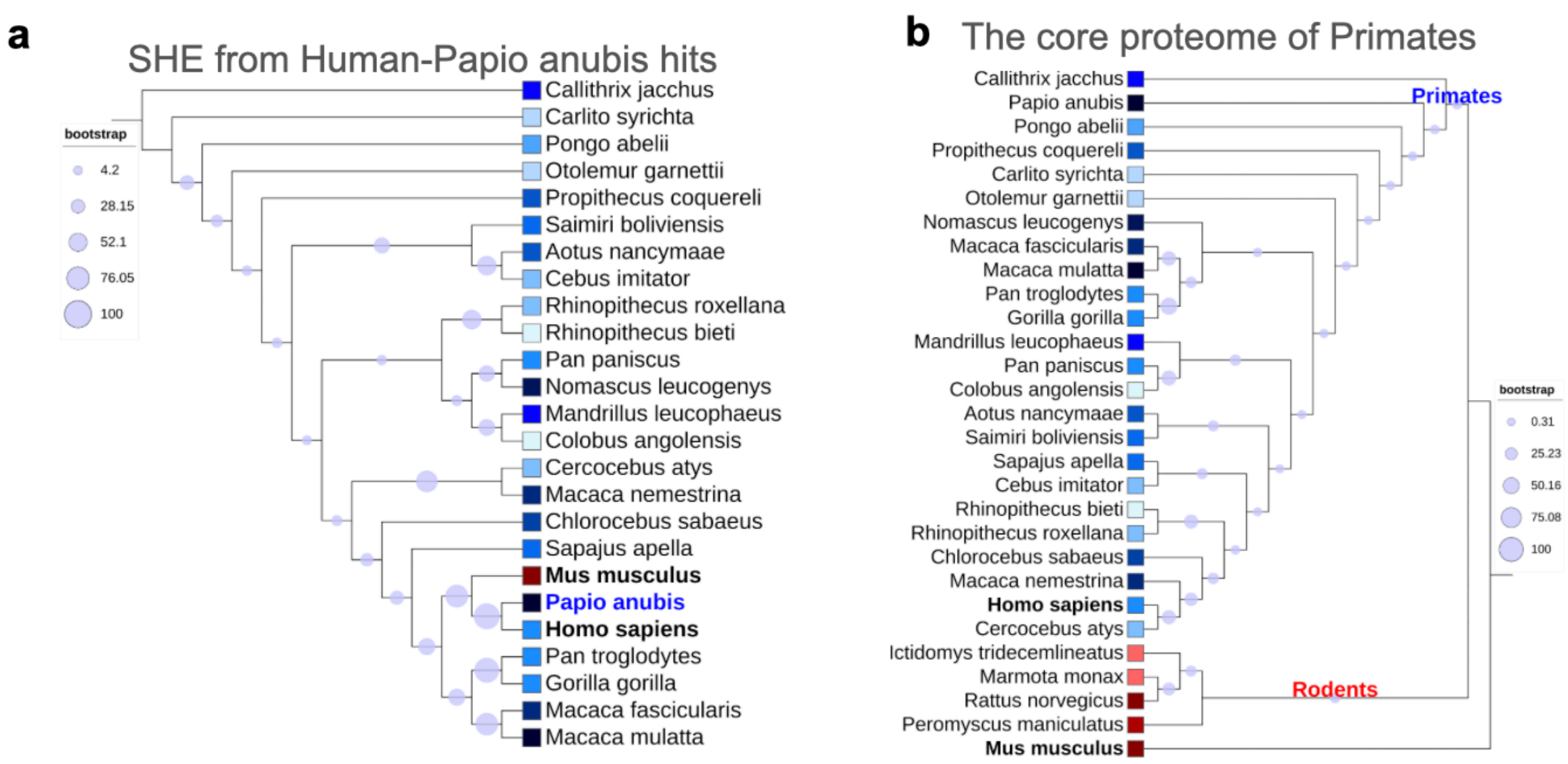

Supplementary Figure 5. SHE, Primates and Rodents.

a) SHE tree pruned to 12,310 *Homo sapiens-Papio anubis* (least proteome completeness among Primates - 64%) protein structural hits. The absence of missing hits drives the sampling of Human and Baboon together, confirming missing proteins as the driving factor in the construction of the tree. Here, the house mouse (*Mus musculus*) is included to further show the weight of no missing proteins in the tree-building method. **b)** SHE tree pruned to 2,773 core proteins among Primates (no missing proteins in Primates). Primates are thus clustered only based on the bitscore values from their structural comparisons. Rodents are now correctly clustered as an independent group because of the greater weight of missing proteins.

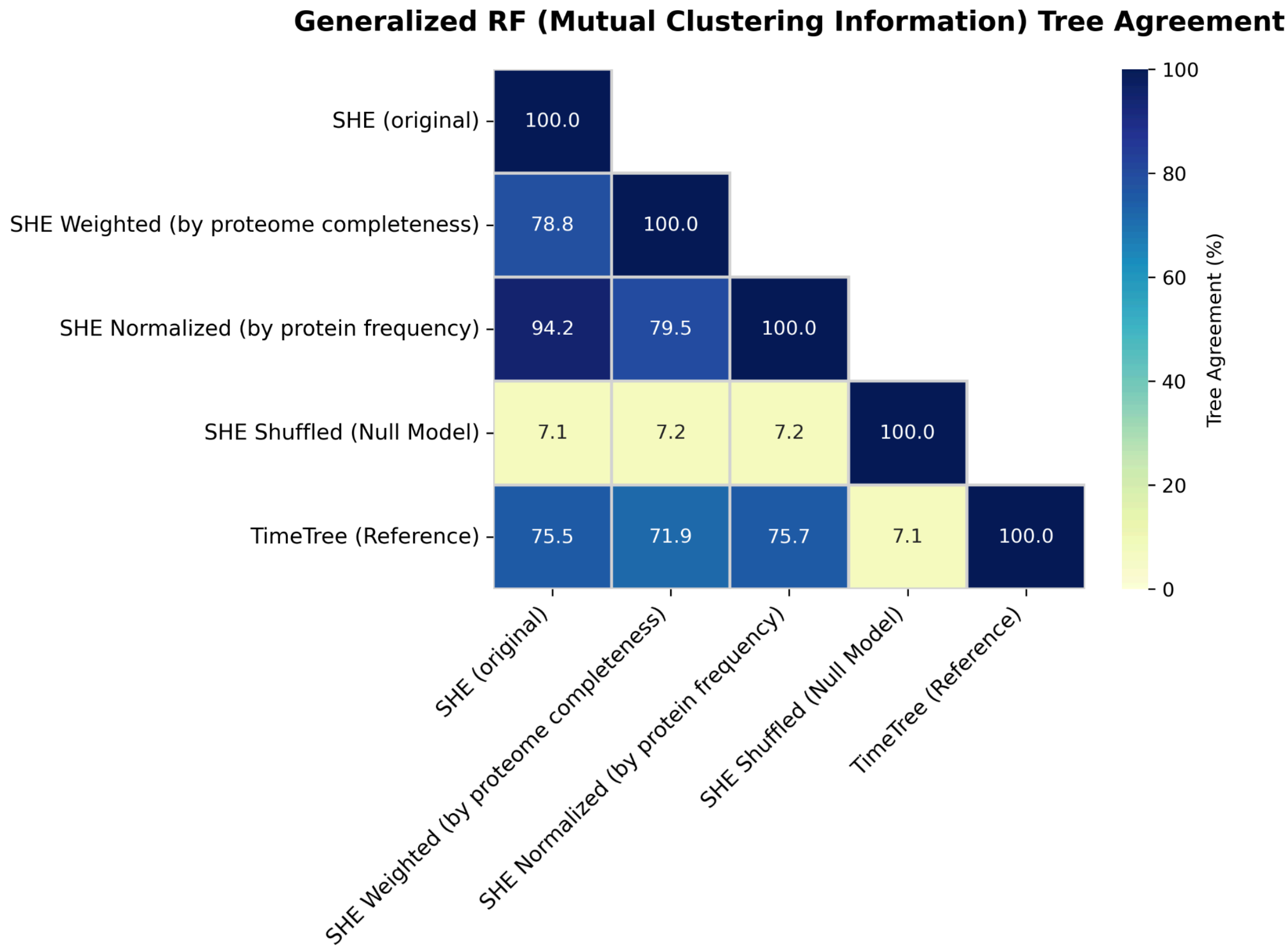

Supplementary Figure 6. Variations of SHE and agreement to reference phylogeny of Eukarya. Comparison between different ways of building the all vs all proteome distance matrix: original, weighting by proteome completeness, normalising on the protein frequency and shuffling the SHE distance matrix. All SHE tree variations were built using NJ and 10,000 bootstraps. TimeTree pruned to 1086 common species to SHE was used as a reference (root at *Acanthamoeba castellanii*).

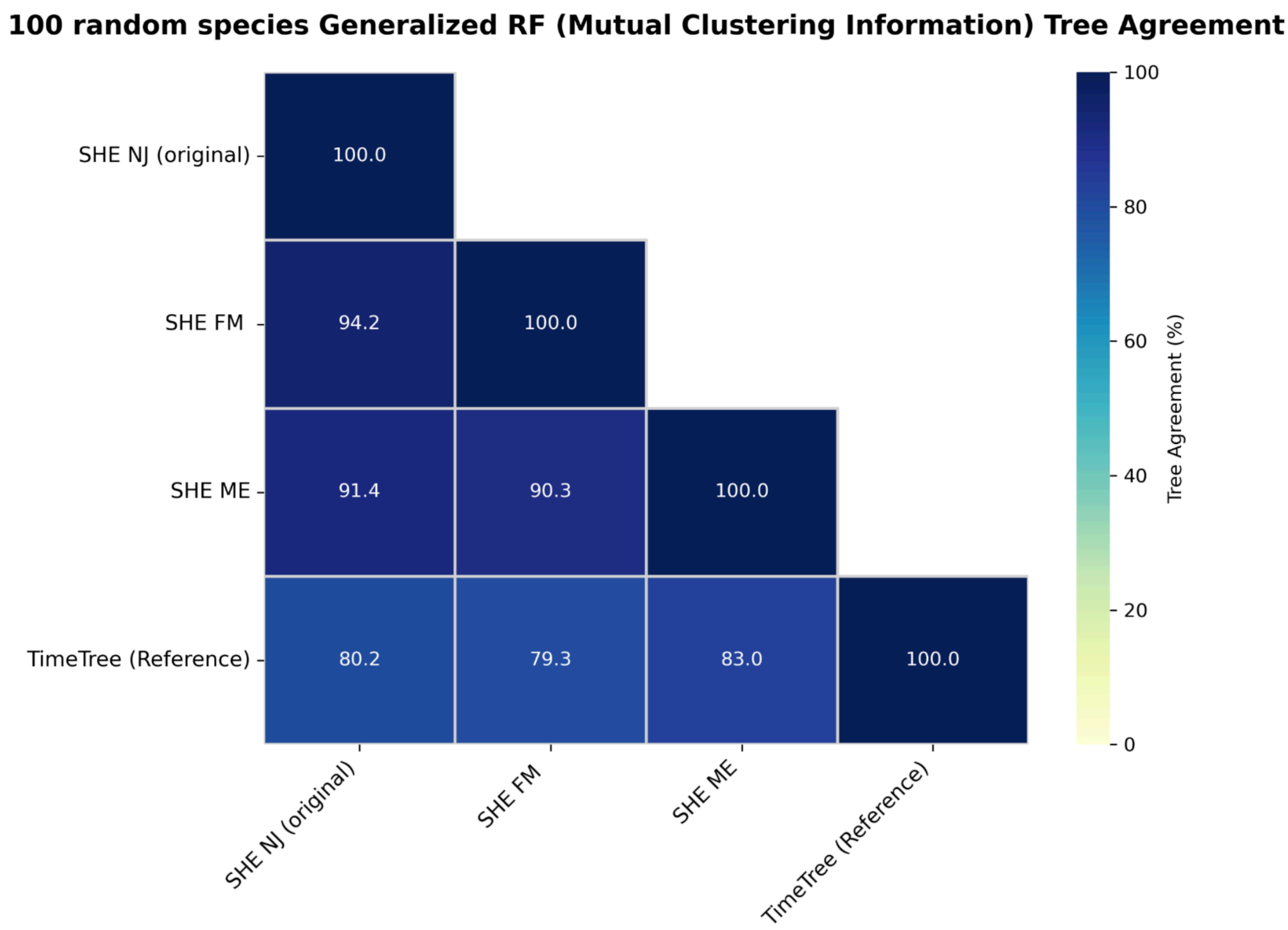

Supplementary Figure 7. Neighbour-joining is robust for building SHE from a proteome-wise distance matrix. Since the SHE pipeline only relies on a proteome-wise distance matrix, the tree could not be built using a standard sequence-based Maximum Likelihood method. Here, 100 species were randomly selected to prove that Neighbour Joining (NJ) is robust at this scale, as shown by very high topological agreement (above 91%) obtained by rebuilding the same tree using **Fitch-Margoliash (FM)** and **Minimum Evolution (ME)** methods (10000 bootstraps). The tree agreement corresponds to 1-normalised mutual clustering information distance. The Timetree built from the same 100 species input was used as reference after pruning to the 79 common species (insufficient data in TimeTree to place the missing 21 taxa) and rooting to *Edaphochlamys debaryana*.

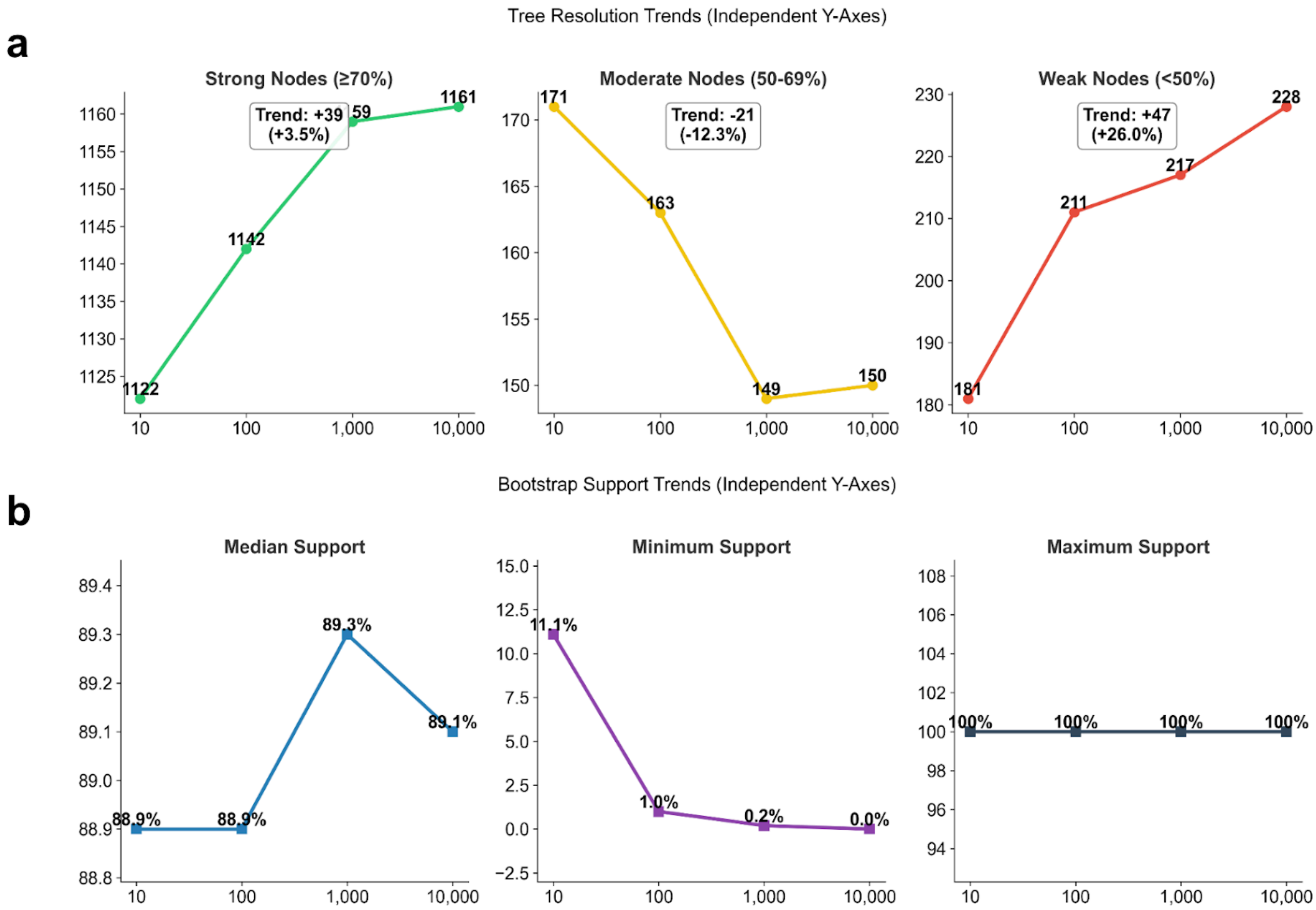

Supplementary Figure 8: Convergence of bootstrap support values with an increasing number of replicates. The analysis was performed with 10, 100, 1,000, and 10,000 replicates. **a)** Trends in phylogenetic resolution categorised by node strength: Strong (≥ 70%), Moderate (50-69%), and Weak (< 50%). Panels are displayed with independent y-axes to highlight relative trends across different magnitudes. This view reveals that while strong nodes remain stable, there is a distinct increase in weak nodes and a decrease in moderate nodes, suggesting a shift from ambiguous to definitively unresolved states as sampling increases. **b)** Plot of key statistical metrics for bootstrap support across all nodes, showing that median and maximum support remain high while minimum support quickly converges to zero.

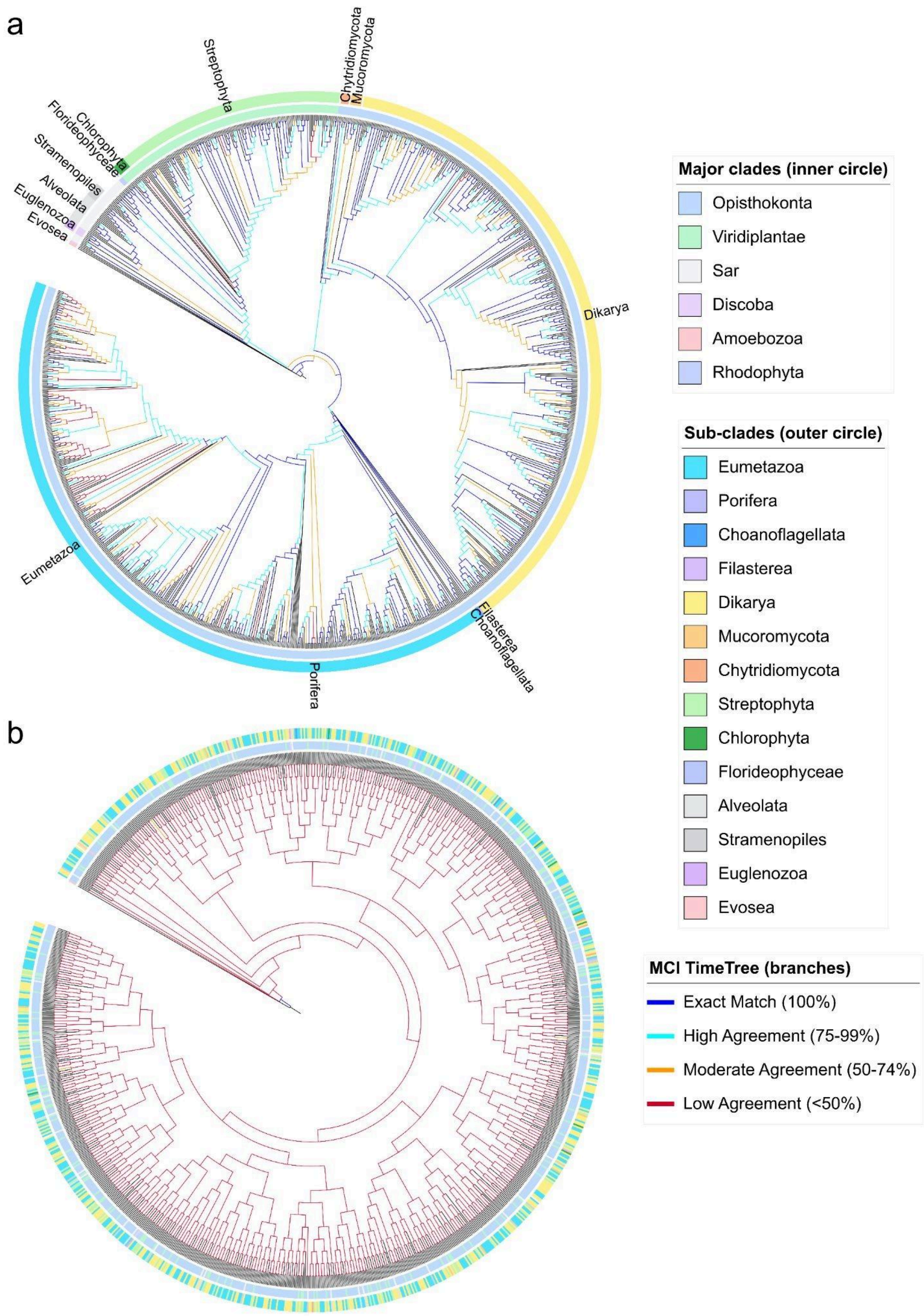

Supplementary Figure 9. SHE branches and clades topology agreement with reference. a) Pruned SHE tree used for comparison to TimeTree. Branch topologies were evaluated using MCI and mapped onto a colour scale ranging from blue (MCI score >=0.999) to red (MCI score <0.50), with cyan and orange intermediates. The inner circle

colours the species based on their major clade, while the outer circle colours are based on selected representative sub-clades. **b)** Pruned shuffled SHE tree compared to TimeTree. Branch topologies were evaluated using MCI and mapped onto a colour scale ranging from blue (MCI score >=0.999) to red (MCI score <0.50), with cyan and orange intermediates. The inner circle colours the species based on their major clade, while the outer circle colours are based on selected representative sub-clades.

Supplementary Tables

Supplementary Table 1: Classification of internal nodes by bootstrap support strength. Node counts are shown for 10, 100, 1,000, and 10,000 replicates. Nodes are stratified into Strong (≥70%), Moderate (50–69%), and Weak (<50%) categories. Total internal nodes indicate the degree of tree resolution relative to the theoretical maximum, demonstrating improved resolution.

Metric	10 Replicates	100 Replicates	1,000 Replicates	10,000 Replicates
Strong Nodes (≥70%)	1122	1142	1159	1161
Moderate Nodes (50-69%)	171	163	149	150
Weak Nodes (<50%)	181	211	217	228
Total Internal Nodes	1474	1516	1525	1539

Supplementary Table 2: Bootstrap support value statistics across replicates. Median, minimum, and maximum support percentages calculated for 10, 100, 1,000, and 10,000 bootstrap replicates.

Metric	10 Replicates	100 Replicates	1,000 Replicates	10,000 Replicates

Median Support	88.90%	88.90%	89.30%	89.10%
Minimum Support	11.10%	1.00%	0.20%	0.00%
Maximum Support	100.00%	100.00%	100.00%	100.00%
